# Effective targeting of breast cancer stem cells by combined inhibition of Sam68 and Rad51

**DOI:** 10.1038/s41388-022-02239-4

**Published:** 2022-02-25

**Authors:** Alice Turdo, Miriam Gaggianesi, Simone Di Franco, Veronica Veschi, Caterina D’Accardo, Gaetana Porcelli, Melania Lo Iacono, Irene Pillitteri, Francesco Verona, Gabriella Militello, Alessio Zippo, Vittoria Poli, Luca Fagnocchi, Sven Beyes, Stefania Stella, Rossano Lattanzio, Naida Faldetta, Vincenzo L. Lentini, Rossana Porcasi, Giuseppe Pistone, Maria Rita Bongiorno, Giorgio Stassi, Ruggero De Maria, Matilde Todaro

**Affiliations:** 1 Department of Health Promotion, Mother and Child Care, Internal Medicine and Medical Specialties (PROMISE), University of Palermo, Palermo, Italy; 2 Department of Surgical, Oncological and Stomatological Sciences (DICHIRONS), University of Palermo, Palermo, Italy; 3 Department of Cellular, Computational and Integrative Biology, University of Trento, Trento, Italy; 4 Department of Clinical and Experimental Medicine, A.O.U. Policlinico-Vittorio Emanuele, Center of Experimental Oncology and Hematology, University of Catania, Catania, Italy; 5 Department of Innovative Technologies in Medicine and Dentistry, Center for Advanced Studies and Technology (CAST), ‘G. d’Annunzio’ University of Chieti-Pescara, Chieti, Italy; 6 Villa Sofia-Cervello Hospital, Palermo, Italy; 7 Dipartimento di Medicina e Chirurgia Traslazionale, Facoltà di Medicina e Chirurgia, Università Cattolica del Sacro Cuore, Roma, Italy; 8 Fondazione Policlinico A Gemelli IRCCS, Roma, Lazio Italy

**Keywords:** Breast cancer, Cancer stem cells

## Abstract

Breast cancer (BC) is the second cause of cancer-related deceases in the worldwide female population. Despite the successful treatment advances, 25% of BC develops resistance to current therapeutic regimens, thereby remaining a major hurdle for patient management. Current therapies, targeting the molecular events underpinning the adaptive resistance, still require effort to improve BC treatment. Using BC sphere cells (BCSphCs) as a model, here we showed that BC stem-like cells express high levels of Myc, which requires the presence of the multifunctional DNA/RNA binding protein Sam68 for the DNA-damage repair. Analysis of a cohort of BC patients displayed that Sam68 is an independent negative factor correlated with the progression of the disease. Genetic inhibition of Sam68 caused a defect in PARP-induced PAR chain synthesis upon DNA-damaging insults, resulting in cell death of TNBC cells. In contrast, BC stem-like cells were able to survive due to an upregulation of Rad51. Importantly, the inhibition of Rad51 showed synthetic lethal effect with the silencing of Sam68, hampering the cell viability of patient-derived BCSphCs and stabilizing the growth of tumor xenografts, including those TNBC carrying *BRCA* mutation. Moreover, the analysis of Myc, Sam68 and Rad51 expression demarcated a signature of a poor outcome in a large cohort of BC patients. Thus, our findings suggest the importance of targeting Sam68-PARP1 axis and Rad51 as potential therapeutic candidates to counteract the expansion of BC cells with an aggressive phenotype.

## Introduction

Breast cancer (BC) is the first malignancy in woman worldwide [1, 2]. Early BC is a curable disease in 80% of patients, as opposed to advanced BC that is characterized by a median overall survival of 2–3 years [1]. Based on estrogen receptor (ER), progesterone receptor (PR), and human epidermal growth factor receptor-2 (HER2) status, differentiated BCs are treated with endocrine therapy or HER2 inhibitors. Triple-negative breast cancers (TNBCs) currently lacks targeted therapies and partially benefit from the use of genotoxic compounds. An exception is made for TNBC patients harboring *BRCA* mutations, who are eligible for the therapeutic regimen based on PARP inhibitors and chemotherapy. Notwithstanding these standard antitumor therapies succeed in reducing disease progression, 35% of BC patients relapse within 10 years [1]. Outstanding advances have been obtained in curing localized BCs, while metastatic disease still lacks effective therapeutic approaches and represents the second cause of cancer-related mortality in women worldwide [3, 4]. Compelling evidence showed that therapy-spared BC cells are endowed with stem-like properties and are responsible for tumor relapse and recurrence [5]. Cancer stem-like cells have been identified and prospectively isolated from BC through the cell surface markers CD44^high^/CD24^low^ and the prominent activity of the detoxifying enzyme aldehyde dehydrogenase 1 (ALDH1) [6, 7]. Mammary gland tissue homeostasis, remodeling and regeneration are finely tuned by adult stem cells, which retain self-renewal and multi-lineage differentiation ability. As a consequence of epigenetic and/or genetic alterations, those cells may acquire a malignant behavior and be in charge of tumor seeding [7]. Peculiarities of normal stem cells are retained by their malignant counterparts, including quiescence, active DNA-repair machinery, expression of ABC drug transporters and constitutive resistance to apoptosis [5].

The oncogene *MYC* is one of the most known stemness transcriptional factors that is also associated with DNA-damage repair [8, 9]. Its role is not restricted to the maintenance of normal stem cells but also involves their neoplastic transformation [8]. Our recent report highlighted that Myc starts an epigenetic reprogramming of breast cells, causing cell dedifferentiation into a stem cell-like state and transcriptional activation of oncogenic pathways [10].

Src associated substrate during mitosis of 68 kDa (Sam68) is a DNA/RNA binding protein involved in a plethora of biological processes of cancer onset and progression [11]. Sam68 retains a heteronuclear ribonucleoprotein particle K homology (KH) domain to control the alternative splicing of several cancer-related gene transcripts, including Bcl-x, Cyclin D1, CD44, SF2/ASF, and Survivin [11–13]. Of note, Sam68 interacts with the splicing activator SRm160 promoting inclusion of v6 exons in CD44 (CD44v6), a marker of metastatic cancer stem cells (CSCs) [12, 14]. Moreover, in cooperation with PARP, a novel role of Sam68 to implement an appropriate DNA-repair mechanism by orchestrating the formation of poly(ADP-ribose) (PAR) polymer has been recently reported in mouse embryonic fibroblasts and colon cancer cells [15].

The possibility to interfere with the DNA-repair mechanism is a promising strategy to face the progression of BCs bearing DNA-repair defects. Indeed, the use of PARP inhibitors proved to target cells harboring an impaired recovery mechanism from DNA errors, such as the inactivation of BRCA [16]. PARP mediates the repair of single-strand DNA filaments and its pharmacological inhibition causes the accumulation of DNA breaks, which are ultimately fixed by the double-strand DNA-repair proteins BRCA [16]. Therefore, BRCA-deficient cancer cells, as well as cancer cells harboring dysfunctions in the homologous recombination (HR) repair, are subjected to unsustainable DNA damage that leads to a “synthetic lethal” cell death [16, 17]. Although PARP inhibitors considerably improved the therapeutic response of BRCA-mutant BC, inter- and intra-tumor heterogeneity limits the treatment efficacy and causes the selection and expansion of the aggressive CSC pool expressing Rad51, responsible for DNA-damage repair *via* HR [18, 19].

Here, we investigated whether DNA repair’s molecular events would underlie the resistance to standard therapy of persistent BC cells. Thus, it became crucial to determine the biomarkers of response to predict the efficacy of DNA-repair inhibitors and to identify effective therapeutic targets.

We demonstrated that the high Myc expression in BC stem-like cells promotes Sam68 transcription, and activation of Rad51 following DNA damage. Combined targeting of Sam68 and Rad51 reduces the cell viability of BC stem-like cells and induces growth stabilization of tumor xenografts. Our data indicate Myc, Sam68, and Rad51 as prognostic biomarkers and promising targetable candidates in BC.

## Results

### Myc regulates the transcriptional activation of Sam68

We previously provided evidence that Myc plays a fundamental role in sustaining the stem-like state of breast cells through the transcriptional activation of genes involved in cell reprogramming and oncogenesis [10]. Due to the small-scale dimension of BC surgical samples available for research studies, patient-derived xenografts (PDX) have been used as a renewable source to obtain larger tumors by serial passage transplantation. This model has also proved to increase the frequency of stem-like cells [20, 21]. Cells directly isolated from PDXs mimic the in vivo tumor heterogeneity and represent a good preclinical model for cancer studies [22].

**Fig. 1:**
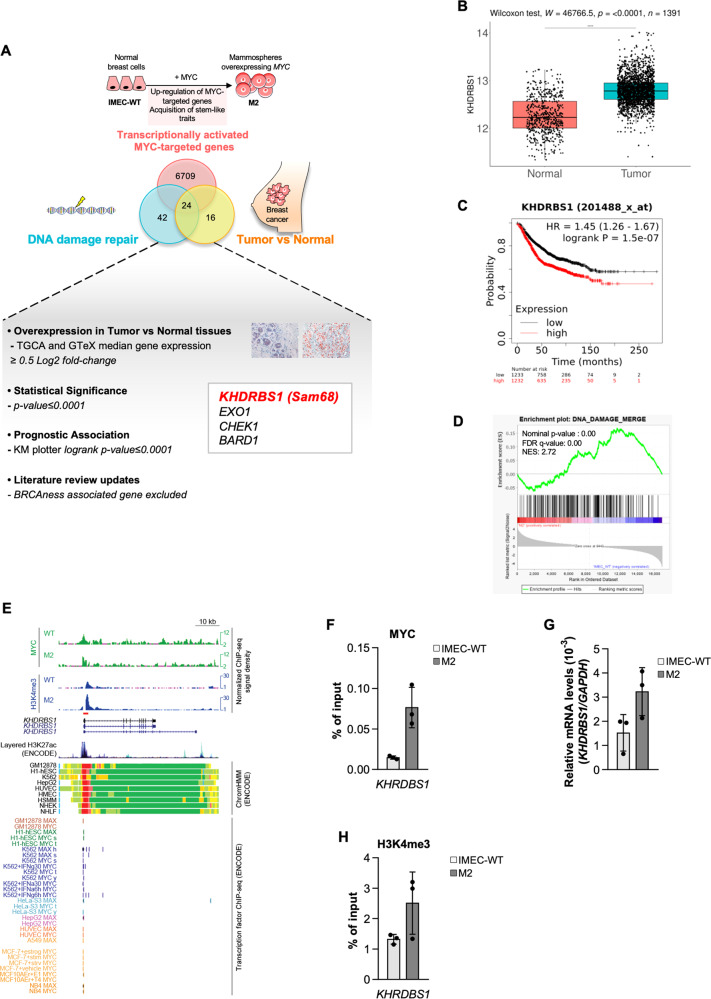
BCSphCs express high levels of Myc. **A** Workflow showing the selection strategy for *KHDRBS1* among DNA-damage response genes transcriptionally activated by Myc and significantly associated to breast cancer prognosis. Venn diagram showing the overlap between Myc-transcriptionally activated genes, DNA-damage response genes and genes associated to breast cancer. Specifically, genes were retrieved from: (i) microarray data of Myc-overexpressing mammospheres (M2) (GSE86407); (ii) published dataset (MD Anderson Human-DNA Repair Genes, https://www.mdanderson.org/documents/Labs/Wood-Laboratory/human-dna-repair-genes.html), BioRad DNA-damage signaling pathway (SAB Target List H96) and recently published DNA-damage-associated genes (Supplementary Table 6); and (iii) breast cancer *versus* normal breast tissues TCGA BRCA and GTeX gene expression data (Supplementary Table 7). Genes were further selected for association to the worse relapse-free survival probability in breast cancer (Supplementary Table 8) [25] and novelty in the field, excluding known genes associated with *BRCAness*. **B** Box plot representing the distribution of log2 gene expression of *KHDRBS1* retrieved from TCGA BRCA (*n* = 1212) and GTeX (*n* = 179) gene expression data (RNASeq2GeneNorm). *p* value was calculated with Wilcoxon rank sum test. **C** Kaplan–Meier plots of relapse-free survival (RFS) probability of BC patients stratified by high or low *KHDRBS1* expression levels. **D** GSEA of DNA-repair gene signatures in IMEC-WT *versus* M2 (*n* =3). **E** Scheme showing MYC and H3K4me3 PCR amplicons localization (red box) on IMEC-WT and M2 cells and layered H3K27ac signals on *KHDRBS1* (*SAM68*) promoter from ENCODE. Chromatin state was assessed by ChromHMM from ENCODE. MYC-MAX binding on multiple cell lines was assessed by ChIP-seq from ENCODE. **F** ChIP-qPCR estimating MYC binding at *SAM68* promoter in IMEC-WT and M2 cells. Data are mean ± SEM (*n* = 3). **G** qRT-PCR analysis of *SAM68* gene expression in IMEC-WT and M2 cells. Data are mean ± SEM (*n* = 3). **H** ChIP-qPCR of H3K4me3 deposition at *KHDRBS1* (*SAM68*) promoter in IMEC-WT and M2 cells. Data are mean ± SEM (*n* = 3).

Breast cancer sphere cells (BCSphCs), were isolated and propagated from three serial transplantation of patient-derived xenograft (PDX) of human luminal and basal BC specimens (Supplementary Fig. SlA and Supplementary Table 1). BCSphCs display an enrichment, at different ratio, in stem-like CD44^high^/CD24^low^ subpopulation and express high levels of other stem cell markers such as *MYC* and *VIMENTIN* (Supplementary Fig. SlA–D), suggesting that this cell compartment has a phenotype closer to mammary basal-myoepithelial stem cells, as compared with BC luminal and TNBC cell lines [23]. Of note, the analysis of a cohort of 21 BCSphC lines showed a significant correlation between CD44^high^/CD24^low^ expression and the mRNA and protein expression levels of MYC (Supplementary Fig. SlE–G). To gain further insight into the involvement of Myc in stemness reprogramming, we investigated the Myc-induced transcriptional changes involving DNA-repair genes, a hallmark of stem-like cells, in normal mammospheres overexpressing *MYC* (Fig. 1A and Supplementary Table 6) (GSE86407) [10, 24]. The overlap with differentially expressed genes in tumor *versus* normal breast tissues (Supplementary Table 7) identified a subset of 24 genes, 4 of which associated to the highest negative prognostic impact in BC (*KHDRBS1*, *EXO1*, *CHEK1*, *BARD1*) [25]. *KHDRBS1* (Sam68) was then selected as an attractive gene for new therapeutic intervention, given the already established contribution of *EXO1*, *CHEK1*, *BARD1* in the DNA-repair machinery, and in particular in the “BRCAness” genomic landscape (Fig. 1B, C) [26–28]. These results are consistent with gene set enrichment analysis (GSEA) showing *SAM68* enrichment among DNA-damage genes in Myc-overexpressing stem-like cells (Fig. 1D and Supplementary Table 8). Additionally, according to the ENCODE Transcription Factor Targets Dataset and confirmed by ChIP-qPCR, Myc appears engaged to the promoter region and bound to *cis*-regulatory elements of *SAM68* (Fig. 1E, F). In line with these results, the increase of *SAM68* mRNA levels was associated with a greater deposition of histone mark H3K4me3 at *SAM68* regulatory elements in cells overexpressing Myc (Fig. 1G, H). Given that Myc-transcriptionally regulates the oncoprotein Sam68, which is overexpressed in several human cancers [11, 29], we selected this gene for further investigation as an unprecedented pivot of BC DNA-damage repair machinery.

### Sam68 expression correlates with breast cancer progression

**Fig. 2:**
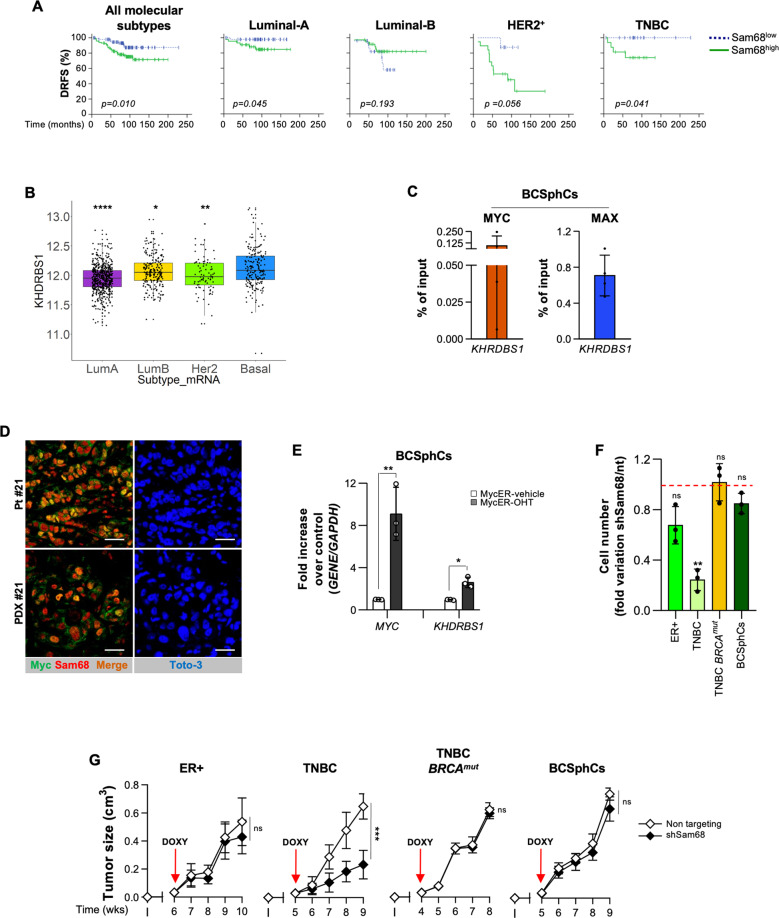
Sam68 is a marker of poor prognosis in breast cancer. **A** Kaplan–Meier plots of distant relapse-free survival (DRFS) of BC patients stratified by high or low Sam68 protein expression levels. Patients were categorized according to all molecular subtypes (*n* = 211) and Luminal-A (*n* = 91), Luminal-B (*n* = 61), HER2^+^ (*n* = 27), TNBC (*n* = 32), HER2^+^ + TNBC (*n* = 59) BCs. **B** Box plot representing the distribution of log2 gene expression of *KHDRBS1* retrieved from TCGA BRCA gene expression data (RNASeq2GeneNorm). *p* value was calculated with Wilcoxon rank sum test. The indicated statistics refer to each molecular subtype *versus* basal subtypes. *p value ≤ 0.05; **p value ≤ 0.01; ****p value ≤ 0.0001. **C** ChIP-qPCR estimating MYC and MAX binding at *SAM68* promoter in BCSphCs (#4 and #15). Data are mean ± SEM of two independent experiment for each BCSphCs. **D** Expression of Myc (green color) and Sam68 (red color) on paraffin-embedded sections on parental BC and corresponding PDX tissue. Nuclei were counterstained with Toto-3 (blue color). Scale bar represents 40 µm. **E** Relative mRNA expression levels of *MYC* and *KHDRBS1* on BCSphCs (#4, #13, and #21) expressing a MycER fusion protein induced by 50 nM of OHT. Data are represented as fold mRNA level changes of OHT-treated cells over vehicle. Data are represented as mean ± SD of three independent experiments. *p value ≤ 0.05; **p value ≤ 0.01. **F** Cell proliferation analysis of ER+ (MCF7), TNBC (BT549), TNBC *BRCA*^*mut*^ (HCC1937) BC cell lines and BCSphCs (#1, #4, #13, and #21) transduced with doxycyclin-inducible non-targeting (nt) and short hairpin Sam68 (shSam68). Data are represented as fold variation of shSam68 over scr. ns not significant; **p value ≤ 0.01. **G** Size of tumors generated by orthotopic injection of ER+ (MCF7), TNBC (BT549), TNBC *BRCA*^*mut*^ (HCC1937) BC cell lines and BCSphCs (#4, #13) in immunocompromised mice (NOD/SCID) at the indicated time points. Data are expressed as mean ± SD (*n* = 5 mice per group). ns not significant, ***p value ≤ 0.001.

We further examined whether the expression Sam68 in the tumor tissue correlated with the clinical outcome of BC. In line with a previous analysis [29], we observed that Sam68 is present at variable intensity in breast tumor cells while being barely detectable in adjacent nontumor breast tissue (Supplementary Fig. S2A–C) and normal tissues (Supplementary Fig. S2C). The analysis of a cohort of 211 primary BCs showed a significant negative correlation between Sam68 expression and distant relapse-free survival (DRFS) probability (Fig. 2A and Supplementary Fig. S2D, E) in Luminal-A and TNBC patients. Importantly, multivariate analysis denoted that Sam68 is an independent negative prognostic factor of DRFS showing the higher statistical significance over the most important clinical parameters (Supplementary Table 4). Immunohistochemistry analysis of the different subtypes indicated that the association between high Sam68 expression and distant relapse occurs independently of tumor size and grade both in Luminal-A patients and when HER2 and TNBC patients are analyzed together, due to the limited size of both cohorts (Supplementary Table 5). Notably, the analysis of a large cohort of BCs (*n* = 1063), stratified according to Sam68 expression levels, highlighted Sam68 expression abundance in undifferentiated BC as compared to the other subtypes, particularly the luminal-A (Fig. 2B).

In order to investigate whether the expression levels of Sam68 are regulated by Myc in BCs, we analyzed BCSphCs and BC cell lines by ChiP-qPCR, which detected both Myc and its co-regulator Max binding at Sam68 promoter (Fig. 2C and Supplementary Fig. 2F). Immunofluorescence and immunohistochemistry analysis revealed that primary BC (patient #21) and its matching patient-derived xenograft (PDX #21) tissues display nuclear co-localization of Myc and Sam68 (Fig. 2D and Supplementary Fig. 2G). Moreover, the ectopic expression of a MycER fusion protein, induced by the 4-hydroxytamoxifen (OHT), significantly enhanced Sam68 expression levels in BCSphCs, while Myc silencing decreased its mRNA levels (Fig. 2E and Supplementary Fig. 2H). These data show a positive correlation between Sam68 and Myc expression in BC.

To investigate the functional role of Sam68 in BC, we silenced Sam68 expression with a lentiviral vector encoding for the doxycycline-inducible short hairpin RNA specific for *SAM68* mRNA (shSam68) [30, 31]. The silencing of *SAM68* significantly reduced the proliferation activity of TNBC BC cell lines. However, ER+ cell lines, TNBC cells harboring *BRCA1* mutation and BCSphCs resulted barely affected by Sam68 downregulation (Fig. 2F and Supplementary Fig. S2I–K). The impaired proliferation promoted by *SAM68* silencing was likely due to the upregulation of cell cycle inhibitor p21 (Supplementary Fig. S2L, M) [29]. To evaluate the therapeutic relevance of targeting Sam68, established BC cell lines and BCSphCs, transduced with doxycycline-inducible shSam68, were allowed to grow in the mammary fat pad of immunocompromised mice. Xenografts generated by *BRCA*^*mut*^ BC cells and BCSphCs did not display growth inhibition following silencing of Sam68 (Fig. 2G and Supplementary Fig. S2N). Of note, the induction of Sam68 silencing was able to delay the growth of tumor xenografts generated by the injection of *BRCA* wt TNBC cell lines, whereas those obtained by ER+ were not significantly influenced, likely due to the different role of Sam68 in tumor development according to the divergent *TP53* mutational status of these cell lines (Fig. 2G and Supplementary Fig. S2N) [32, 33]. To investigate whether that the concomitant inactivity of Sam68 and TP53 arrested cancer cell growth, *TP53* wild-type MCF7 cell lines were engineered by using CRISPR/Cas9 technology to introduce a *TP53* mutation (R248Q). *TP53*-mutation in combination with Sam68 silencing affects the cell growth of engineered MCF7 cells (Supplementary Fig. S2O–S).

### Myc confers resistance to DNA-damaging agents through Rad51 regulation in breast cancer cells

**Fig. 3:**
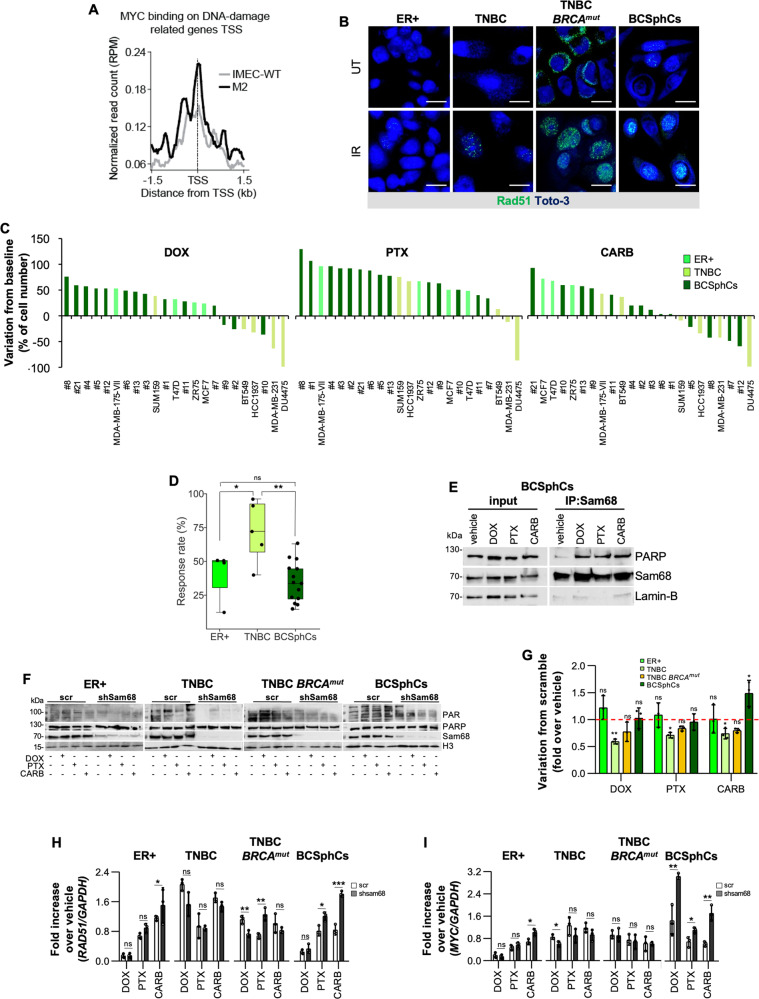
Breast cancer sphere cells are chemoresistant and show activation of the HR pathway. **A** MYC binding on DNA-damage related genes transcription start sites (TSS) on IMEC-WT and M2 breast cells. **B** Representative immunofluorescence analysis of Rad51 foci formation in ER+ (MCF7), TNBC (BT549), TNBC *BRCA*^*mut*^ (HCC1937) BC established cell lines and BCSphCs (#4) untreated (UT) and after 6 h of 8 Gy single dose γ-irradiation (IR). Nuclei were counterstained by Toto-3 (blue). Scale bar represents 10 µm. **C** Waterfall plot analysis of doxorubicin (DOX, 200 nM, *left panel*), paclitaxel (PTX, 10 nM, *middle panel*) and carboplatin (CARB, 100 µM, *left panel*) response at 72 h in ER+ and TNBC BC established cell lines and BCSphCs. **D** Response rate distribution to chemotherapy for ER+ and TNBC BC established cell lines and BCSphCs treated as in (**C**). Middle line shows the median value of response per group, while single points represent the average value of BC cell response to DOX, PTX and CARB. Data are mean of three independent experiments. Statistical analysis was performed by using Kruskal–Wallis test. Ns not significant, *p value ≤ 0.05; **p value ≤ 0.01. **E** Immunoblot analysis of PARP and Sam68 (input) and after immunoprecipitation (IP) with Sam68 antibody in BCSphCs (#15) treated for 4 h with vehicle, doxorubicin (DOX), paclitaxel (PTX) and carboplatin (CARB). Lamin-B was used as loading control. **F** Immunoblot analysis of nuclear PAR, PARP, and Sam68 in scramble (scr) and short hairpin Sam68 (shSam68) ER+ (MCF7), TNBC (BT549), and TNBC *BRCA*^*mut*^ (HCC1937) BC cell lines and BCSphCs (#4) treated with vehicle, doxorubicin (DOX), paclitaxel (PTX) and carboplatin (CARB) for 4 h. H3 was used as loading control. **G** Cell proliferation analysis of ER+ (MCF7), TNBC (BT549), and TNBC *BRCA*^*mut*^ (HCC1937) BC cell lines and BCSphCs (#1, #4, #13, #21) transduced with scramble and short hairpin Sam68 (shSam68) treated with vehicle, doxorubicin (DOX), paclitaxel (PTX) and carboplatin (CARB) for 72 h. Data are represented as fold variation of shSam68 over scramble. Data are mean ± SD of three independent experiments. ns not significant; *p value ≤ 0.05; **p value ≤ 0.01. **H**, **I** Relative mRNA expression levels of *RAD51* (H) and *MYC* (I) on scramble (scr) and short hairpin Sam68 (shSam68) ER+ (MCF7), TNBC (BT549), and TNBC *BRCA*^*mut*^ (HCC1937) BC cell lines and BCSphCs (#12 and #13) treated with vehicle, doxorubicin (DOX), paclitaxel (PTX), and carboplatin (CARB) for 24 h. Data are represented as fold mRNA level changes of treated scr and shSam68 cells over vehicle. Data are represented as mean ± SD of three independent experiments. Ns not significant, *p value ≤ 0.05; **p value ≤ 0.01; ***p value ≤ 0.001.

We next sought to evaluate whether the oncogene *MYC*, one of the key regulators of *SAM68* transcription and already known to be involved in the DNA-damage response (DDR) [9, 34], could have a critical impact in the resistance to standard therapies by inducing the recruitment of genes that facilitate DNA-repair. We previously reported that cancer progression could be attributed to the emergence of a remnant therapy-resistant cancer stem-like population, which is characterized by highly efficient activation of DDR [35–37]. We observed that Myc positively modulated genes involved in single and double-strand DNA-repair process, which was paralleled by an enhanced capability of TNBC *BRCA*^*mut*^ BC cells and BCSphCs to accumulate nuclear Rad51 foci following exposure to 8 Gy of radiation therapy (Fig. 3A, B and Supplementary Fig. S3A, B). Moreover, BCSphCs resulted inherently more resistant to chemotherapy than ER^+^ and TNBC established cell lines, regardless the cell cycle status, as confirmed by comet-assay analysis and H2AX staining (Fig. 3C, D and Supplementary Fig. S3C–E).

Of note, cells surviving chemotherapy displayed high expression of *MYC* levels (Supplementary Fig. S3F, G). Recent evidence pointed out that upon genotoxic stress Sam68 interacts with PARP and activates DDR [15, 38]. We hypothesized that Myc-driven Sam68 expression influences the capability of PARP to trigger DDR. Immunoprecipitation analysis showed that Sam68 forms nuclear complexes with PARP in BC cell lines and BCSphCs treated with doxorubicin (DOX), paclitaxel (PTX) or carboplatin (CARB) (Fig. 3E and Supplementary Fig. S3H–I). Moreover, genetic inhibition of *SAM68* caused a defect in PARP-induced PAR chain synthesis following exposure to standard chemotherapy (Fig. 3F and Supplementary Fig. S3J). This phenomenon ultimately resulted in a superior sensitization to chemotherapy of TNBC cells, without affecting the ER^+^ BC cells (Fig. 3G), suggesting that the presence of a *TP53* mutation could render TNBC cells more vulnerable to Sam68 knockdown and the chemotherapy-induced DNA-damage [33, 39]. Even though derived from BCs with different molecular subtypes, BCSphCs were unresponsive to both chemotherapy and *SAM68* silencing and characterized by a significant increase of *RAD51* and *MYC* expression (Fig. 3G–I). Indeed, after *SAM68* knockdown, BCSphCs responded to chemotherapy with a similar increase in HR activity as control cells (Supplementary Fig. S3K).

Collectively, these data indicate Sam68 as a key molecular target for the DDR.

### Rad51 inhibition sensitizes breast cancer sphere cells to Sam68 depletion

**Fig. 4:**
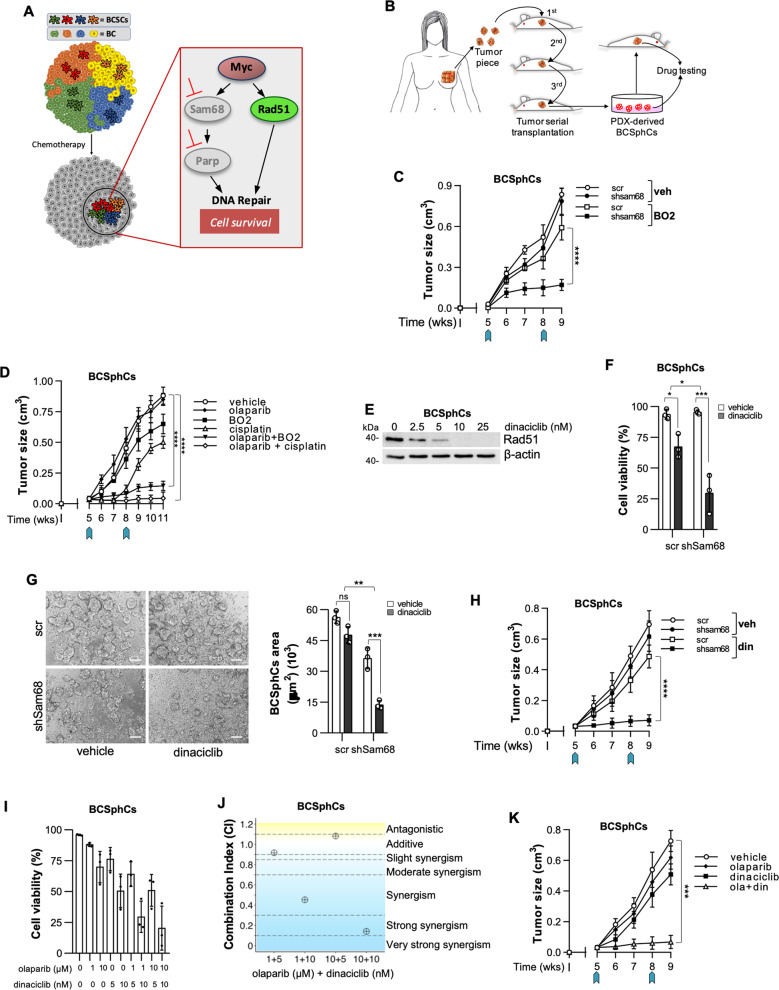
Combined inhibition of Sam68 and Rad51 counteracts the growth of breast cancer sphere cells. **A** Schematic model of DNA-repair signaling pathways mediating the resistance of BC stem-like cells to chemotherapy. **B** Workflow of purification of sphere cells from serially transplanted BC PDX and their use for in vitro and in vivo drug toxicity testing. **C** Size of tumors generated by orthotopic injection of scramble (scr) and short hairpin Sam68 (shSam68) BCSphCs treated with vehicle (veh) and BO2. Arrows indicate the start and the end of treatment. Data are expressed as mean of tumors generated by the injection of BCSphCs (#4, #13, and #21) ± SEM (*n* = 5 mice per group). **D** Size of tumors generated by orthotopic injection of scramble (scr) and short hairpin Sam68 (shSam68) BCSphCs (#4, #13, #21) treated with vehicle, olaparib, BO2, cisplatin and olaparib *plus* BO2 and olaparib *plus* cisplatin and BO2. Arrows indicate the beginning and the end of treatment. Data are expressed as mean of tumors generated by the injection of BCSphCs (#4, #13, and #21) ± SEM (*n* = 5 mice per group). ****p value ≤ 0.0001. **E** Immunoblot analysis of Rad51 in BCSphCs (#15) treated with dinaciclib for 24 h at the indicated concentration. Β-actin was used as loading control. **F** Cell viability percentage of scramble (scr) and short hairpin Sam68 (shSam68) BCSphCs (#4, #13, #15, and #21) treated with vehicle and dinaciclib (10 nM) for 6 days. Data are represented as mean ± SEM (*n* = 2). *p value ≤ 0.05; ***p value ≤ 0.001. **G** Representative images (*left panel*) and quantification of area (*right panel*) of BC sphere cells (#21), transduced with scramble (scr) and short hairpin Sam68 (shSam68) lentiviral vectors, treated with vehicle and dinaciclib for 6 days. Data are represented as mean ± SEM (*n* = 3). Ns not significant, **p value ≤ 0.01; ***p value ≤ 0.001. Scale bar represents 100 µm. **H** Size of tumors generated by orthotopic injection of scramble (scr) and short hairpin Sam68 (shSam68) BCSphCs treated with vehicle (veh) and dinaciclib (din). Arrows indicate the start and the end of treatment. Data are expressed as mean of tumors generated by the injection of BCSphCs (#4, #7, #13) ± SEM (*n* = 5 mice per group). ****p value ≤ 0.0001. **I** Cell viability percentage of BCSphCs (#4, #13, #14, #15, #21) treated with vehicle, olaparib and dinaciclib, alone or in combination, at the indicated concentrations for 6 days. Data are represented as mean ± SD (*n* = 3). **J** Synergy plot representing the combination index (CI), computed in CompuSyn by using Chou-Talalay method, for each olaparib and dinaciclib dose pair, calculated from cell viability data of BCSphCs (#13). **K** Size of tumors generated by orthotopic injection of BCSphCs treated with vehicle, olaparib, dinaciclib and olaparib plus dinaciclib. Arrows indicate the start and the end of treatment. Data are expressed as mean of tumors generated with BCSphCs (#4, #7, #13) ± SEM (*n* = 5 mice per group). ***p value ≤ 0.001.

In order to render BCSphCs vulnerable to the inhibition of Sam68-PARP1 axis, we sought to target the synthetic lethal partner Rad51 (Fig. 4A). The use of BCSphCs purified from serially transplanted PDX provides a powerful platform for high-throughput drug sensitivity screens (Fig. 4B) [21]. BCSphCs were treated with the specific activity inhibitors of Rad51, BO2 and RI-1, which promoted a considerable induction of cell death in combination with the knockdown of *SAM68* (Supplementary Fig. S4A). Sam68 downregulation together with BO2 treatment resulted in a considerable size reduction in xenograft tumors generated by the injection of BCSphCs and TNBC *BRCA*^*mut*^ BC cells (Fig. 4C and Supplementary Fig. S4B). In the attempt to study whether the pharmacological inhibition of the Sam68-PARP1 axis, instead of the *SAM68* knockdown, was able to make BCSphCs sensitive to small molecules targeting Rad51, we exposed cells to the PARP inhibitor olaparib. Whilst olaparib promoted only a modest decrease in cell viability as a single agent, the combination with BO2 and RI-1 resulted in a considerable growth inhibition in BCSphCs (Supplementary Fig. 4C). Cisplatin has been described as one of the most effective chemotherapeutic regimens in reducing tumor burden of TNBCs [40]. Given the side effects commonly reported in cisplatin-treated patients, due to drug non-specific targeting [33, 41], we sought to compare the efficacy of olaparib in combination with Rad51 inhibitor or cisplatin. Notwithstanding olaparib *plus* cisplatin halted tumor growth, mice experienced a 15–20% reduction in body weight loss (Fig. 4D and Supplementary Fig. S4D–H). Of note, olaparib and BO2 induced long term disease stabilization without causing any sign of mice sufferance (Fig. 4D and Supplementary Fig. SD–H). These data point out the therapeutic relevance of Rad51 and Sam68 inhibition in highly aggressive BC subtypes. Several clinical trials, including the use of dinaciclib, have been designed to obtain Rad51 indirect targeting (NCT01434316, NCT01676753, NCT00732810, NCT01357395). Dinaciclib is an inhibitor of the cyclin-dependent kinases (CDKs) that are involved in the transcription of DNA-repair genes including *RAD51* and *BRCA1* (Fig. 4E and Supplementary Fig. S4I–K) [9, 33, 42]. In accordance with the effect exerted by small molecules targeting Rad51, TNBC *BRCA*^*mut*^ BC cells and BCSphCs, bearing the knockdown of *SAM68*, were sensitive to dinaciclib (Fig. 4F and Supplementary Fig. S4L). Upon treatment with dinaciclib, BCSphCs defective for Sam68 displayed an attenuated capacity to form sphere structures, while the viability of healthy cells was only slightly affected (Fig. 4G and Supplementary Fig. S4M). In line with in vitro data, dinaciclib significantly reduced the size of tumor xenografts generated by orthotopic injection of shSam68 BCSphCs and TNBC *BRCA*^*mut*^ BC established cell line (Fig. 4H and Supplementary Fig. S4N). Immunohistochemical analysis of tumor xenografts showed that dinaciclib potently lowered the expression of CD44 and Rad51 in *SAM68* down-regulated cells (Supplementary Fig. S4O).

Notably, olaparib in combination with dinaciclib reduced the growth rate of TNBC *BRCA*^*mut*^ cells and BCSphCs, sparing normal breast cells (Fig. 4I, J and Supplementary Fig. S4P–R) [43]. Moreover, olaparib-based combination therapy suppressed the formation of Rad51 foci and induced a growth stabilization of BCSphCs and TNBC *BRCA*^*mut*^ tumor xenografts, which lacked regrowth after treatment suspension (Fig. 4K and Supplementary Fig. S4S, T). Of note, mice treated with dinaciclib and olaparib did not show any signs of sufferance as demonstrated by normal morphology of hepatocytes and minor variation of body weight (Supplementary Fig. S4U–X).

Taken together, these data indicate the inhibition of Sam68-PARP1 axis and Rad51 as potential therapy to counteract the expansion of BC cells with an aggressive phenotype.

### Sam68 together with Myc and Rad51 identifies an aggressive molecular subtype of breast cancers

Endocrine therapy is the preferred choice for ER+ patient treatment. Chemotherapy drugs are eventually administered to the 25% of BC patients who relapse following endocrine therapy within 10 years [1, 44]. However, most of these patients also show resistance to DNA-damaging chemotherapy [42]. It has been demonstrated that BCs surviving endocrine therapy are enriched in a subpopulation of cells with stem-like characteristics that do not express ER [45].

**Fig. 5:**
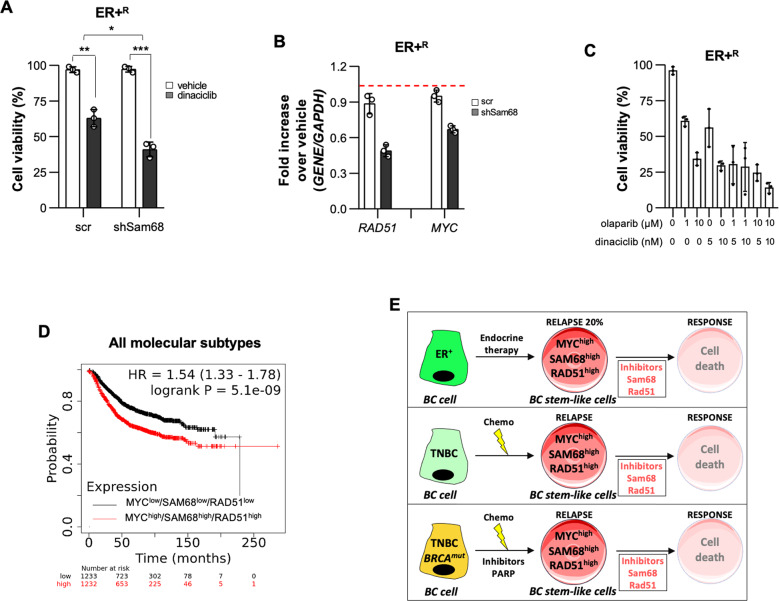
Myc, Sam68 and Rad51 characterize breast cancers with poor clinical outcome. **A** Cell viability percentage of scramble (scr) and short hairpin Sam68 (shSam68) ER+^R^ (MCF7) BC cell line treated with vehicle and dinaciclib (10 nM) for 6 days. Data are represented as mean ± SEM (*n* = 4). *p value ≤ 0.05; **p value ≤ 0.01; ***p value ≤ 0.001. **B** Relative mRNA expression levels of *RAD51* and *MYC* on scramble (scr) and short hairpin Sam68 (shSam68) ER+^R^ (MCF7) BC cells treated with vehicle and dinaciclib for 6 days. Data are represented as fold mRNA level changes of treated scr and shSam68 over vehicle (*n* = 3). **C** Cell viability percentage in ER+^R^ (MCF7) BC cells treated with vehicle, olaparib and dinaciclib, alone or in combination, at the indicated concentrations for 6 days. Data are represented as mean ± SD (*n* = 3). **D** Kaplan–Meier plots of relapse-free survival (RFS) probability of BC patients of all molecular subtypes stratified by high or low *MYC*, *KHDRBS1*, and *RAD51* expression levels. **E** Schematic model showing the persistence of a BC stem-like population, characterized by high expression levels of *MYC, SAM68*, and *RAD51*, following standard anticancer therapies.

We observed that ER+ BC cells resistant to tamoxifen (ER+^R^) show the upregulation of *MYC* (Supplementary Fig. S5A). Notably, ER+, as well as ER+^R^ BC cells exhibited a significant reduction of cell viability after treatment with dinaciclib, whose efficacy was further increased by silencing of *SAM68* (Fig. 5A and Supplementary Fig. S5B, C).

In ER + ^R^ BC cell population, the treatment with dinaciclib as single agent decreases the levels of *RAD51* and *MYC*, which were maintained low by the shSam68 (Fig. 5B and Supplementary Fig. S5D). We also noticed more than threefold decrease of stem-related genes in shSam68 ER+^R^ BC cells treated with dinaciclib (Supplementary Fig. S5E). Accordingly, cell viability was significantly hampered by the exposure to the combination of olaparib and dinaciclib in ER+ and ER+^R^ BC cells (Fig. 5C and Supplementary Fig. S5F, G). These results indicate that Sam68-PARP axis could play a fundamental role in governing the resistance of ER+ cells to endocrine therapy.

We next investigated the clinical relevance of the magnitude of *MYC, SAM68* and *RAD51* association as prognostic biomarkers. Gene expression signature characterized by *MYC*^*high*^, *SAM68*^*high*^, and *RAD51*^*high*^ was associated with worse relapse-free survival in a cohort of 2465 BC patients, having more prognostic significance than individual gene expression (Fig. 5D and Supplementary Fig. S5H, I). These data suggest that undifferentiated and differentiated BC resistant to standard therapies, and thereby with a poor clinical outcome, could be clustered in a *MYC*/*SAM68/RAD51* high signature (Fig. 5E).

## Discussion

Here, we demonstrated that all molecular subtypes of BCs harbor a subpopulation of therapy-resistant cells that express high levels of Myc, Sam68 and Rad51, whose targeting effectively inhibit the cell survival. In line with these findings, analysis of a large cohort of BC led to the identification of a signature in which high levels of Sam68, Myc, and Rad51 identify a particular aggressive subset of tumors. Patients affected by BC have a 35% risk of developing locoregional or distant recurrence within 10 years of breast-conserving surgery, regardless the cancer cell differentiation [44]. This frequency can be reduced by the administration of radiation therapy or endocrine therapies in the adjuvant setting [44]. Despite the efficacy of these treatments, the risk of BC recurrence and death is no <20% [44]. Current anticancer treatments are unable to target heterogeneous cancer cell population in advanced tumors. Both genetic and epigenetic heterogeneity contribute to spare from therapies the tumorigenic population of stem-like cells, which are characterized by high activity of repairing genotoxic damage. Cancer sphere cells are three-dimensional structures, being characterized by an enrichment of cancer stem-like cells [21, 46, 47]. Given its heterogeneous composition, this model may represent a useful tool that can be exploited to a predict anticancer treatment response [21, 22]. In this context, in addition to our first observation regarding the capability of Myc to confer stem-like traits and oncogenic reprogramming in BCs [10], we herein show that Myc regulates the transcription of *KHDRBS1* (*SAM68*), a DNA-damage repair gene, which is involved in the resistance of BCSphCs to common anticancer drugs.

A multivariate analysis of BC tissue microarrays showed that the most aggressive molecular subtypes, highly-expressing Sam68, are positively associated with recurrence and metastasis rate. Based on data demonstrating that poorly differentiated BCs display higher content of stem-like cells than differentiated cancers [23], we deem that Sam68 is a fundamental cornerstone for this cell compartment. Small molecules targeting Sam68, such as CWP232291 [48], has been already considered for phase I clinical studies in myeloma (NCT02426723) and in acute myeloid leukemia and myelodysplastic syndrome (NCT01398462) patients, posing this molecule as a safe and efficacious compound to sensitize relapsed and refractory neoplasia to standard therapies [49]. The closely related compound CWP232228 has been demonstrated to compromise the transcription of known Wnt pathway target genes by inducing the formation of a complex between Sam68 and the CBP binding partner, thus avoiding CBP/β-catenin interaction selectively in CSCs [50]. Thus, these results suggest that targeting Sam68 is a clinically feasible approach for targeting CSCs in diverse type of oncological diseases. The capability of Sam68 to repair the DNA has been linked to PARP activity [15]. Sam68 enables PARP to form PAR polymer, which in turn activate a plethora of antiapoptotic genes through the nuclear translocation of NF-κB p65 subunit [15, 38]. Accordingly, after genotoxic stress, BCSphCs show the nuclear interaction between Sam68 and PARP. Likewise, Sam68-induced PAR production mediates the single-strand DNA-repair of BC stem-like cells treated with chemotherapy through the recruitment of ATM, ATR and DNA-dependent protein kinase [15, 38]. Although the genetic downregulation of *SAM68* combined with chemotherapy significantly reduced PAR formation, BCSphCs reached an incomplete antitumor response, which was associated with a potent increase of *MYC* and *RAD51* expression levels. In contrast, the cell death induced by Sam68 targeting and chemotherapy, in TNBC established cell lines, could be likely due to the presence of an already impaired DNA-repair machinery conferred by *TP53* and/or *BRCA* mutations in the non stem population [33, 37, 51]. ER+ BC cells were not significantly influenced, likely due to the different role of Sam68 in tumor development according to the divergent *TP53* mutational status of these cell lines [32, 33].

Dinaciclib is an inhibitor of CDK 1, 2, 5, 9, and 12 that impairs the transcription of DNA-repair genes including *RAD51*. In the last years, dinaciclib has demonstrated a potential antitumor activity for the treatment of solid cancers including BC (NCT01676753; NCT00732810; NCT01434316). Our data provide evidence that Rad51 targeting significantly reduces the viability of BCSphCs silenced for *SAM68*, required as co-activator of PARP and synthetic lethal partner of Rad51. Treatment with dinaciclib, in combination with conventional chemotherapy, showed substantial toxicities jeopardizing the entering in phase II studies of metastatic TNBC patients [52]. However, a double regimen based on dinaciclib and a therapy specific for Sam68 might result in a reduced toxicity to normal organs and improved therapeutic efficacy.

Rad51 sustains the cell survival of BCSphCs, posturing this molecule as a biomarker of response to PARP inhibitors [18]. These compounds also showed efficacy in *BRCA* wild-type BCs harboring dysfunction in the HR pathway, a phenomenon known as “BRCAness” [17]. These findings corroborate our data on the efficacy of Sam68-PARP axis and Rad51 inhibition in BC stem-like cells, regardless of the *BRCA* mutational background.

We also show that ER+ BC cells, resistant to endocrine therapy, are enriched in cells expressing BC stemness markers, including *MYC*. These refractory cells likely upregulate DNA-damage repair genes, whose targeting by dinaciclib alone was not sufficient to counteract the cell growth [42]. Nevertheless, Sam68 targeting significantly reduced the cell viability of tamoxifen-resistant ER+ BC cells treated with dinaciclib, suggesting that this combination may result in a potential effective therapy.

Taken together these data suggest that BCs, across the molecular subtypes, harbor a subpopulation of therapy-resistant stem-like cells that emerges during tumor relapse. Thus, the *MYC/SAM68/RAD51* signature could stratify patients for prognosis and predict for a more effective targeted therapy.

## Material and methods

### Breast cancer cells isolation, culture, and treatment

BC specimens were provided by the University Hospital “P. Giaccone” and the Hospital “Ospedali Riuniti Villa Sofia-Cervello”, in accordance with the ethical standards of the Institutional Committee responsible for human experimentation. Specimens’ staging was established according to the AJCC classification of malignant tumor 7th Edition. Samples were classified into different molecular subtypes evaluating the immunohistochemical markers ER, PR, HER2, and Ki-67 [53] (Supplementary Table 1).

In order to obtain BCSphCs from PDX models, BC specimens were cut in four pieces of 1–2 mm^3^ and serially transplanted for three passages into the mammary fat pad of 4–6 weeks old female NOD/SCID mice (Charles River Laboratories), in presence of matrigel (BD Biosciences). After the third passage, PDX specimens were collected and digested with collagenase (1.5 mg/mL, Gibco) and hyaluronidase (20 mg/mL, Sigma-Aldrich). Subsequently, single-cell suspensions were allowed to grow as spheres in non-adherent conditions and in serum-free stem cell medium (SCM) supplemented as described in [22]. This culture condition favors the enrichment of cells endowed with stem-like properties [20, 46]. The BCSphCs area was calculated with Image J software. Established BC cell lines were purchased from CLS cell line service (MCF7, ZR75, T47D, BT549) and DMSZ (HCC1937) and propagated according to manufacturer recommendations. SUM159 cell line was kindly provided by Prof. Max Wicha (University of Michigan, Ann Arbor, MI) and cultured in Ham’s F-12 supplemented with 5% FBS, 1 μg/mL hydrocortisone (Sigma-Aldrich) and 5 μg/mL insulin (Sigma-Aldrich). The MDA-MB-436 cell line was cordially provided by Valeria Coppola (Istituto Superiore di Sanità, Italy) and cultured in DMEM/F-12 (Corning), 10% FBS (Corning) and 5 μg/mL insulin (Sigma-Aldrich). The authentication of BCSphCs, their related tumor tissues and established BC cells was performed by using a highly informative short tandem repeat (STR) system (GlobalFiler PCR amplification Kit; Applied Biosystems) and analyzed by ABIPRISM 3130 genetic analyzer (Applied Biosystems). BCSphCs and BC cell lines were tested for mycoplasma infection every 3 months using the MycoAlert^TM^ Plus Mycoplasma Detection Kit (Lonza). BCSphCs and BC cells were treated with doxorubicin (200 nM, Sigma-Aldrich), paclitaxel (10 nM, Sigma-Aldrich), carboplatin (100 µM, Sigma-Aldrich), BO2 (10 µM, Callbiochem), RI-1 (20 µM, Callbiochem), dinaciclib (2.5, 5, 10, and 25 nM, Selleckchem) and olaparib (1 and 10 µM, Selleckchem), alone or in combination for up to 6 days. Chemotherapy drugs were replenished every 48 h, whereas olaparib every 72 h. Synergy plots represented combination index (CI) were computed in CompuSyn using Chou-Talalay method by treating each cell line with different olaparib and dinaciclib dose pairs and evaluating cell viability. A CI < 1 represented different levels of synergism (slight, moderate, strong, very strong), otherwise it indicated additivity (CI = 1) or antagonism (CI > 1) between two drugs [54]. Scatter plots were generated in R with ggplot2 package. To obtain tamoxifen-resistant ER+ (ER+^R^) cells, MCF7 cells were treated with stepwise dose increase of tamoxifen (Sigma-Aldrich) up to 6 µM. When cells’growth was not inhibited with 6 µM of tamoxifen, ER + ^R^ cells were established and maintained in culture in presence of 6 µM tamoxifen. Irradiation (8 Gy) of BCSphCs and BC cells was performed using a Caesius source.

### RNA extraction and gene expression analysis

Total RNA of BCSphCs and BC cell lines was purified using TRIzol (Thermo Fisher Scientific) and RNA concentration was determined with NanoDrop™ 1000 Spectrophotometer (Thermo Fisher Scientific). For gene expression analysis, 1 µg of total RNA, after genomic DNA removal, was retrotranscribed and analyzed with a PrimePCR custom panel (BioRad) according to the manufacturer’s instructions. For single assay, total RNA was retrotranscribed using the High-Capacity cDNA Reverse Transcription Kit (Applied Biosystems) and qRT-PCR was performed using the following primers: *MYC* (HS00153408_m1), *KHDRBS1* (HS00173141_m1), *RAD51* (HS00947967_m1), and *GAPDH* (Hs02786624_g1) (Applied Biosystem) and *BRCA1* (For CTGAAGACTGCTCAGGGCTATC, Rev AGGGTAGCTGTTAGAAGGCTGG) and *GAPDH* (For GCTTCGCTCTCTGCTCCTCCTGT, Rev TACGACCAAATCCGTTGACTCCG). The mRNA level was normalized to *GAPDH* housekeeping gene and calculated using the comparative CT method (ΔΔCt method).

### DNA extraction and next-generation sequencing

Total DNA was isolated from BCSphCs using the DNeasy Blood & Tissue Kits (Qiagen) and the quantification of DNA obtained was performed using the Qubit ds DNA HS assay Kit (Invitrogen) by Qubit 2.0 Fluorometer (Invitrogen) according to manufacturer’s instructions. For the library preparation, 30 ng of DNA from each sample was used. Library preparation carried out manually according to the standard Ion AmpliSeq protocol (Thermo Fisher Scientific) with validated community panel of 24 amplicons designed to analyze all coding exons of the *TP53* gene (Ion AmpliSeq™ TP53 Panel) according to manufacturer’s instructions. After amplification, the two pools were combined manually, and we created the barcoded gDNA libraries according to protocol (Ion P1 Adapter and Ion Express barcode, Thermo Fisher Scientific). Subsequently, the libraries were purified using AMPure XP magnetic beads (Beckman Coulter). The concentration of each library was measured using Ion Ampliseq Library TaqMan Quantitation kit (Thermo Fisher Scientific) and 40 pM of each TP53 library were combined in an equimolar ratio in the Ion Chef™ Library Sample Tube (barcoded tube) and loaded onto the Ion Chef™ Instrument. The sequencing was performed with Ion550 Chip by using Ion Gene Studio S5 Plus instrument (Thermo Fisher Scientific). Reads were aligned to GRCh37 (hg19) human reference sequence. The reported mutations were analyzed by Ion Reporter software (Thermo Fisher Scientific) and validated in the International Agency for Research on Cancer (IARC) TP53 database (www-p53.iarc.fr), ClinVar (https://www.ncbi.nlm.nih.gov/clinvar/) and Cosmic (https://cancer.sanger.ac.uk/cosmic) to confirm the assigned class of the mutation (Supplementary Table 2). The pathogen and benign predictions of variants were further investigated by bibliographic research. The *TP53* mutational status of BC cell lines were retrieved from COSMIC Cell Lines Project v94 (released 28-MAY-21) compared with literature data [32, 55] (Supplementary Table 3).

### Chromatin immunoprecipitation (ChIP) and ChIP-qPCR

Human mammary cells wild-type (IMEC-WT) and IMEC cells overexpressing MYC and cultured in ultralow conditions as mammospheres (M2) [10] were fixed adding formaldehyde to the cell culture media to a final concentration of 1%, for 10 min at room temperature (RT). To quench the reaction, glycine (125 mM) was added for 5 min at RT. ChIP procedure was performed as previously described [10]. Briefly, cells were resuspended in lysis buffer (50 mM Tris-HCl pH 8, 0.1% SDS, 10 mM EDTA pH 8, 1 mM PMSF, protease inhibitor cocktail) and chromatin was sonicated (Bioruptor Pico sonicator, Diagenode) for 4 cycles of 30 s, to reach an average fragment size of ~300 kb. 10 µg of sonicated chromatin was incubated overnight at 4 °C with 4 µg of MYC (sc-764 Santa Cruz Biotechnology), MAX (sc-197 Santa Cruz Biotechnology) and trimethyl histone H3 Lys4 (H3K4me3, rabbit polyclonal, Millipore) antibodies. Previously, protein G-coupled Dynabeads (Thermo Fisher Scientific) were blocked overnight at 4 °C with 1 mg/ml sonicated salmon sperm DNA (Thermo Fisher Scientific) and BSA (1 mg/ml). Blocked G-coupled Dynabeads were incubated with the ChIP reactions for 4 h at 4 °C. Then, magnetic beads were recovered and sequentially washed with ice-cold RIPA buffer (10 mM Tris-HCl, pH 8, 0.1% SDS, 1 mM EDTA, pH 8, 140 mM NaCl, 1% DOC, 1% Triton, 1 mM PMSF, protease inhibitor cocktail), ice-cold RIPA-500 buffer (10 mM Tris-HCl, pH 8, 0.1% SDS, 1 mM EDTA, pH 8, 500 mM NaCl, 1% DOC, 1% Triton, 1 mM PMSF, protease inhibitor cocktail), ice-cold LiCl buffer (10 mM Tris-HCl, pH 8, 0.1% SDS, 1 mM EDTA, pH 8, 250 mM LiCl, 0.5% DOC, 0.5% NP40, 1 mM PMSF, protease inhibitor cocktail) and TE buffer (10 mM Tris-HCl, pH 8, 1 mM EDTA, pH 8, 1 mM PMSF, protease inhibitor cocktail). Finally, the crosslinking was reversed in elution buffer (10 mM Tris-HCl, pH 8, 0.5% SDS, 5 mM EDTA, pH 8, 300 mM NaCl) at 65 °C overnight and DNA purified using Agencourt AMPure XP SPRI beads (Beckman) and dissolved in 60 ml of Tris-HCl, pH 8.0. DNA was analyzed by quantitative PCR using the SYBR Green ER qPCR SuperMix Universal kit (Thermo Fisher Scientific). All experimental values were shown as percentage of input and the values obtained with a nonimmune serum (background) were subtracted to the relative ChIP signals.

### Tissue microarray, immunohistochemistry and immunofluorescence analysis

Tissue microarrays (TMA) were constructed by removing 2-mm diameter cores of histologically confirmed invasive breast carcinoma (*n* = 211) areas from each original paraffin block and re-embedding these cores into gridded paraffin blocks, using a precision instrument (MTA, Beecher Instruments) (Supplementary Tables 4, 5). After antigen retrieval (pH 6.0), 5 μm sections were incubated overnight at 4 °C with Sam68 antibody (rabbit polyclonal, Santa Cruz Biotechnology). The anti-rabbit EnVision kit (Agilent) was used for signal amplification. Slides were evaluated by pathologist without knowledge of the clinicopathological data. Immunoreactivity for Sam68 in tumor cells was nuclear with a concomitant positive nuclear staining of stromal cells present in most cases. Sam68 expression was quantified as percent of immunoreactive tumor cells. To dichotomize Sam68 expression, a cutoff value of 91% of positive cells was chosen, corresponding to the 50th percentile. Thus, tumors with >91% of positive cells (*n* = 105) were considered Sam68^High^, and those with ≤91% of positive cells (*n* = 106) were considered Sam68^Low^. Pathologic tumor size and tumor grade, as well as ER, PR and Ki-67 expression were dichotomized according to the St. Gallen criteria (2013). HER2 membranous staining was scored according to Herceptest (Agilent) and classified as positive if the intensity was scored 3+, with more than 30% of cells showing complete membrane staining, or if the intensity was scored 2+ in the presence of amplification of the HER2 gene as assessed by fluorescent in situ hybridization.

For immunohistochemistry analysis, 5 μm-thick paraffin-embedded sections of BC tissues, their normal counterpart and tumor xenografts were heated in a retrieval solution (pH 6.0) for antigen unmasking, permeabilized with PBS plus 0.1% Triton X-100 (TBS) for 10 min on ice and exposed overnight at 4 °C to Sam68 antibody (C-20, rabbit IgG, Santacruz Biotechnology), CD44 (156-3C11, mouse IgG2a, Cell Signaling Technology), γH2AX (Ser139, mouse IgG_1_, JBW301, Merk-Millipore), Rad51 (D4B10 rabbit IgG, Cell Signaling Technology) and Myc (rabbit polyclonal, Cell Signaling Technology). Sections were incubated with biotinylated immunoglobulins, exposed to streptavidin and stainings were revealed using 3-amino-9-ethyl carbazole (AEC, Agilent) substrate and nuclei counterstained with aqueous hematoxylin (Sigma-Aldrich). Immunohistochemical analysis were quantified with Image J—Color inspector 3D. Standard protocols have been used for H&E staining.

For immunofluorescence analysis, BCSphCs and BC cells were centrifugated on cytospin slides or cultured on round coverslips into 24 well plates and fixed with 2% paraformaldehyde for 20 min at 37 °C. After permeabilization, cells were stained overnight at 4 °C using antibodies against Rad51 (D4B10 rabbit IgG, Cell Signaling Technology), Myc (rabbit polyclonal, Cell Signaling Technology) and Sam68 (C-20, rabbit IgG, Santacruz Biotechnology) at appropriate dilutions. Then, cells were labeled with Alexa Fluor 488-conjugated secondary antibodies (Thermo Fisher Scientific) and nuclei counterstained using Toto-3 iodide (Thermo Fisher Scientific). Stainings were analyzed using a confocal microscope (Nikon D-Eclipse C1).

### Cell viability

To evaluate cell proliferation, Cell Titer-Glo Luminescent Cell Viability Assay Kit (Promega) was used according to the manufacturer’s instruction and luminescence was measured by using Infinite F500 (Tecan). Cell viability was assessed by using Trypan blue exclusion assay.

### Immunoprecipitation and western blot analysis

BCSphCs and BC cells were harvested by scraping in ice-cold PBS and resuspended in ice-cold F buffer (10 mM Tris-HCL, 50 mM NaCl, 30 mM sodium pyruvate, 50 nM NaF, 5 µM ZnCl2) (Sigma-Aldrich) freshly supplemented with protease inhibitor cocktail (Sigma-Aldrich), phosphatase inhibitor cocktail 2 and 3 (Sigma-Aldrich), 0.1 nM sodium orthovanadate (Sigma-Aldrich), 10 mM sodium butyrate (Sigma-Aldrich) and 1 mM PMSF (Sigma-Aldrich). To obtain the nuclear fraction, cells were suspended in hypotonic buffer (10 mM HEPES, 10 mM KCl, 10 mM NaCl, 0.1 mM EDTA, 0.1 mM EGTA, 0.5 mM PMSF, 1 mM DTT, 0.6% NP40, protease and phosphatase inhibitor cocktails) and centrifugated at 11,000 × *g* for 30 s. The obtained nuclear pellet was dissolved in hypertonic buffer (20 mM HEPES, 20% glycerol, 400 mM NaCl, 1 mM EDTA, 1 mM EGTA, 0.5 mM PMSF, 1 mM DTT, 0.6% NP40, protease and phosphatase inhibitor cocktails). For immunoprecipitation experiments, an equal amount of protein lysates was incubated overnight at 4 °C with 2 µg of anti-Sam68 antibody (H-4, mouse IgG_1_, Santa Cruz Biotechnology) mixed with protein G-Sepharose (Sigma-Aldrich). The 10% of whole nuclear cell lysates (input) and protein complexes were loaded in SDS-PAGE gels and blotted onto nitrocellulose membranes. Membranes were blocked in 5% non-fat dry milk 0.1% Tween 20 for 1 h at RT and then exposed overnight at 4 °C to Sam68 (rabbit polyclonal, Santa Cruz Biotechnology), PARP (rabbit polyclonal, Cell Signaling Technology), Lamin-B (rabbit polyclonal, Abcam), PAR (10H, mouse IgG3, Abcam), H3 (rabbit polyclonal, AbCam), Rad51 (D4B10, rabbit IgG, Cell Signaling Technology), p21 (12D1, rabbit IgG, Cell Signaling Technology), Myc (rabbit polyclonal, Cell Signaling Technology) and β-actin (8H10D10, mouse IgG2b, Cell Signaling Technology). Primary antibodies were revealed using anti-mouse or anti-rabbit HRP-conjugated (goat IgG; Thermo Fisher Scientific) and detected using Amersham imager 600 (GE Healthcare).

### Production of lentiviral particles and cell transduction

2 × 10^5^ BC cells and BCSphCs were transfected with 20 pmol of specific siRNA targeting c-myc (si-cmyc) and a control siRNA (si-scr) (Origene) using Lipofectamine 2000 (Thermo fisher). Two different siRNA were used in all the experiments.

2 × 10^5^ BC cells and BCSphCs were transfected with the pBABE-MycER vector [56], using XtremeGENE HP DNA Transfection Reagent (Roche). To induce the expression of MycER, cells were treated with 50 nM 4-hydroxytamoxifen (OHT) (Sigma-aldrich) for 48 h.

To produce lentiviral particle HEK-293T packaging cells were transfected with pLK0.1 (scr) and pLK0.1-*KHDRBS1* (shSam68), kindly provided by Prof. Claudio Sette, p-TWEEN LUC-No GFP, inducible non-targeting (Dharmacon) and inducible *KHDRBS1* shRNA (Dharmacon) together with psPAX2 (Addgene, 12260) and pMD2.G (Addgene, 12259) using XtremeGENE HP DNA Transfection Reagent (Roche). Lentiviral particles were concentrated with Lenti-X Concentrator reagent (Clontech) and added to BCSphCs and BC cells in presence of 8 μg/mL of polybrene (Sigma-Aldrich). Cells transduced with pLK0.1 and inducible lentiviral vectors were selected adding 2 µg/mL puromycin (Sigma-Aldrich) in fresh medium every 2 days. ShSam68 was induced by treating cells for 72 h with 1 µg/ml doxycycline (Sigma-Aldrich).

### Comet assay

To analyze the single and double-strand DNA break induced by chemotherapeutic drugs, 1 × 10^5^ BC and BCSphCs were treated with doxorubicin (200 nM, Sigma-Aldrich), paclitaxel (10 nM, Sigma-Aldrich), carboplatin (100 µM, Sigma-Aldrich) for 24 h and alkaline comet assay was performed following the manufacturer instruction (CometAssay^®^ Kit, Trevigen). Tail length was obtained by measuring from the center of the head to the end of the tail of 100 cells.

### HR assay

BCSphCs and BC cells were transfected with pDRGFP (Life Science Market) and pCBASceI (Life Science Market) plasmids and after 72 h the SceI-induced HR was determined by measuring GFP-positive signals by flow cytometry.

### Flow cytometry and cell sorting

BCSphCs and BC cells were washed in PBS twice, stained for 1 h at 4 °C with conjugated antibodies for CD44 (G44-26, mouse IgG2b; BD Biosciences) and CD24 (ML5, mouse IgG2a; R&D Systems) or corresponding isotype-matched controls IgG2b-PE (BD Biosciences) and IgG2A-APC (R&D Systems) and analyzed by using Accuri C6 (BD Biosciences) flow cytometer. To measure cellular DNA content, cells were washed with PBS and incubated with Nicoletti Buffer for 16 h at 4 °C. Enrichment of CD44^high^/CD24^low^ and CD44^high^/CD24^high^ and OFP-positive subpopulations were accomplished by FACSMelody cell sorter. Collected cells were resuspended in PBS with 2% BSA and 2 mM EDTA and filtered with 70 µm mesh. To verify the purity of the obtained subpopulations we performed a post sorting acquisition. Dead cell exclusion was performed by adding 7-AAD (BD Biosciences) or propidium iodide (PI) (Sigma-Aldrich) to cell staining.

### Animals and tumor models

All the in vivo experiments were performed according to the ARRIVE and animal care committee guidelines of the University of Palermo (Italian Ministry of Health authorization n. 56/2020PR and 1280/2015-PR). 2 × 10^6^ Scr and shSam68 BC cells, transduced with luciferase (LUC)-expressing lentiviral vector, were suspended in 50 µl of matrigel (BD Biosciences) 1:6 in SCM and injected into the mammary fat pad of 4–6 weeks old female NOD/SCID mice (Charles River Laboratories). Tumor growth was monitored weekly with a caliper and volume was calculated according to the formula: largest diameter × (smallest diameter)^2^ × π/6. In vivo cell spreading was monitored by the detection of bioluminescence intensity using a Photon IMAGER (Biospace Lab), after the injection of VivoGlo Luciferin (150 mg/kg, Promega). For the experiments performed with BC cells transduced with non-targeting and shSam68 inducible lentiviral vector, doxycycline (2 mg/ml) was added to drinking water when tumor became palpable (0.01–0.03 cm^3^) and replenished every 72 h.

Palpable tumors were treated by intraperitoneal injection with BO2 (Selleckchem, 50 mg/kg, 3 days/week) dissolved in 20% cremophor (Sigma-aldrich), olaparib (Selleckchem, 50 mg/kg, 5 days/week) dissolved in 10% (2-Hydroxypropyl)-β-cyclodextrin, cisplatin (Selleckchem, 4 mg/kg, at day 1, 4, and 7) [41, 43, 57, 58] and dinaciclib (Selleckchem, 25 mg/kg, 3 days/week) dissolved in 20% (2-Hydroxypropyl)-β-cyclodextrin (Sigma-aldrich), alone or in combination for 3 weeks.

### Gene set enrichment analysis (GSEA)

GSEA was performed using previously published expression data for IMEC and M2 cells (GSE86407) and GSEA version 4.0.3. To obtain a comprehensive gene list for DNA damage-associated genes, published and curated datasets (MD Anderson Human-DNA Repair Genes, https://www.mdanderson.org/documents/Labs/Wood-Laboratory/human-dna-repair-genes.html, BioRad DNA-damage signaling pathway (SAB Target List H96) and recently published DNA damage-associated genes were merged to obtain a final list of 325 DNA-damage genes. This list, among others derived from the GSEA database (www.broadinstitute.org/gsea/), were used to elucidate the enrichment of these genes in the two cell lines.

### Statistical analysis

Relapse-free survival (RFS) probability analysis of BC patients (*n* = 2465) stratified by high or low MYC, KHDRBS1 (SAM68) and RAD51 expression levels was performed by Kaplan–Meier plotter tool [25]. BC patients were stratified by high and low expression of *CDC25A*, *CDC25C*, *KHDRBS1*, *PCNA*, and *PPM1D*, and high and low mean expression of *KHDRBS1*, *MYC* and (*BRCC5*) *RAD51* by comparing quartile 1 versus quartile 4. All probe sets for each gene were used.

TCGA BRCA and GTeX gene expression data (RNASeq2GeneNorm) in tumor *versus* normal breast tissues and TCGA BRCA *KHDRBS1* expression data (RNASeq2GeneNorm), grouped by molecular subtypes [59], were downloaded using the UCSC Xena (xenabrowser.net) and retrieved with R and “TCGAbiolinks” and “TCGAWorkflowData”. Data were normalized with the upper quartile method and converted to log2. Otherwise indicated, statistical analysis was performed by Student *t* test and considered a *p* value > 0.05 statistically not significant (ns).

## Supplementary information

Supplementary Figure and Table legends

Supplementary Figure 1

Supplementary Figure 2

Supplementary Figure 3

Supplementary Figure 4

Supplementary Figure 5

Supplementary Table 1

Supplementary Table 2

Supplementary Table 3

Supplementary Table 4

Supplementary Table 5

Supplementary Table 6

Supplementary Table 7

Supplementary Table 8

## Data Availability

All data generated or analyzed during this study will be included in the published article (and its Supplementary Information files). The data that support the findings of this study will be available from the corresponding author upon reasonable request.
