# Supplementary material for: Effective targeting of breast cancer stem cells by combined inhibition of Sam68 and Rad51
Source: Oncogene. 2022 Feb 25;41(15):2196–209. doi: 10.1038/s41388-022-02239-4 (PMC8993694; doi:10.1038/s41388-022-02239-4)
Supplement: Supplementary file 1 — Supplementary Figure and Table legends [file 41388_2022_2239_MOESM1_ESM.pdf]

SUPPLEMENTARY INFORMATION

SUPPLEMENTARY FIGURE LEGENDS

Supplementary Figure 1. BCSphCs express high expression levels of Myc

A. Workflow showing the establishment of BCSphC cultures following serial transplantation of BC PDXs in immunocompromised mice. Scale bar represents 200 µm. **B**. Percentage of $CD44^{high}/CD24^{low}$ BCSphCs (#1-#21) assessed by flow cytometry. **C.** Representative flow cytometry profiles of CD44 and CD24 on the indicated BCSphCs. **D**. Clustergrams of up- and down-regulated genes involved in epithelial-to-mesenchymal transition and stemness in ER+ (MCF7, ZR75, MDA-MB-175VII) and TNBC (MDA-MB-231, BT549, MDA-MB-436) BC cell lines and BCSphCs (#4, #13, #17). **E.** mRNA expression levels of *MYC* on ER+ (MCF7, ZR75, MDA-MB-175VII) and TNBC (MDA-MB-231, BT549), TNBC/$BRCA^{mut}$ (MDA-MB-436, HCC1937) BC cell lines and BCSphCs (#1-#21). Data were normalized to *GAPDH* expression levels. **F**. mRNA expression levels of *MYC* in the indicated BCSphC (#1-#21) subpopulations. GAPDH was used as control. ****p-value≤0.0001, **G**. Representative pre-sorting and post-sorting analysis of BCSphCs (#12) analyzed for CD44 and CD24 expression (*left panel*). Representative immunofluorescence analysis of Myc in post-sorted BCSphCs (#12) (*right panel*).

Supplementary Figure 2. Sam68 is a negative prognostic marker in breast cancer

A. Representative immunohistochemical analysis of Sam68 in BC specimen and its adjacent-normal counterpart. Scale bar represents 20µm. **B.** Immunoblot analysis of Sam68 in 3 representative breast tumor specimens (T) and adjacent-normal counterparts (AN). β-actin was used as the loading control (*upper panel*). Relative protein expression levels of Sam68 in 3 representative BC specimens and adjacent-normal counterparts. Data are mean ± SD (n=3) (*lower panel*). **C.** Box plot showing *SAM68* mRNA levels in normal tissues and BC samples (R2 platform, http://r2.amc.ne, n=1304, p=0). **D.** Representative immunohistochemical analysis of Sam68 high or low expression levels in BC tissues. Scale bar represents 20 µm. **E.** Box-and-whisker diagram of the percent of BC cells, in BC patient specimens (n=155), showing positive staining for Sam68. The median value is showed with a solid line. **F.** ChIP-qPCR estimating MYC and MAX binding at *KHDRBS1* promoter in ER+ (MCF7), TNBC (BT549), TNBC $BRCA^{mut}$ (HCC1937). Data are mean ± SEM of two independent experiment for each BC cell line. **G.** Immunohistochemical analysis of MYC (brown) and Sam68 (brown) in paraffin-embedded sections of parental BC and corresponding PDX tissues. Scale bar represents 40µm. **H.** Relative mRNA expression levels of *MYC* and *KHDRBS1* on BCSphCs (#4, #13 and #21) transduced with two different c-myc siRNAs (si-cmyc). Data are

represented as fold mRNA level changes of si-cmyc over si-ctr cells. Data are represented as mean ± SD of three independent experiments. **I.** Relative band densities of immunoblots for Sam68 in normal breast (NB) cell line (HMEC) and ER+ (MCF7, T47D, MDA-MB-175VII) and TNBC (HCC1937, DU4475, BT549, MDA-MB-231, MDA-MB-436) BC cell lines and BCSphCs (#1, #4, #17, #20). β-actin was used as loading control. Data are mean ± SD (n=3). **J.** Representative image of confocal microscopy analysis of Sam68 (green) in BCSphCs #1. Nuclei were counterstained by Toto-3 (blue). Scale bar represents 40µm. **K.** *SAM68* mRNA level in ER+ (MCF7) BC cell line transduced with doxycycline-inducible non-targeting and short hairpin Sam68 (shSam68) clone 7, 8 and 9 vectors. Clone 9 has been selected for performing *in vitro* and *in vivo* experiments. Data are normalized to *GAPDH* expression levels. Data are means ± SD (n=3). **L.** Relative *SAM68* mRNA levels (*upper panel*) and Sam68 protein expression (*lower panel*) in ER+ (MCF7), TNBC (BT549) and TNBC $BRCA^{mut}$ (HCC1937) cell lines and BCSphCs (#1) transduced with scramble (scr) and short hairpin Sam68 (shSam68). *mRNA* data were normalized to *GAPDH* expression levels. β-actin was used as the loading control. **M.** Immunoblot analysis of Sam68 and p21 in cells as in (L). β-actin was used as loading control (*left panel*). Relative band densities of immunoblots for Sam68 and p21 (*right panel*). **N.** Representative immunohistochemical analysis of Sam68 and isotype-matched control (IMC) of tumor xenografts generated by orthotopic injection of doxycycline-inducible non-targeting and short hairpin Sam68 (shSam68) ER+ (MCF7) BC cell line. Scale bar represents 20 µm. **O**. Gene editing strategy for the knock-in of $TP53^{R248Q}$ mutations based on the use of CRISPR/Cas9 Nuclease vector with OFP reporter and the specific donor DNA. **P**. Flow cytometry analysis showing the MCF7 OFP-positive cell population selected for sorting. **Q**. Electropherograms showing the nucleotide sequence of interest in the DNA of cells carrying the indicated mutations. **R**. Relative *SAM68* mRNA levels (upper panel) and Sam68 protein expression (lower panel) in MCF7 cell lines transduced with scramble (scr) and short hairpin Sam68 (shSam68). mRNA data were normalized to GAPDH expression levels. β-actin was used as the loading control. **S**. Cell proliferation analysis of wild type and *TP53*-mutant MCF7 cells, transduced with scr and shSam68,. Ns, not significant, **p-value≤0.01.

Supplementary Figure 3. BCSphCs treated with chemotherapy show up-regulation of *MYC* and *RAD51*

A. Box plot showing Myc binding on DNA-damage related genes TSS on IMEC-WT and M2 cells, based on ChIP-seq data shown in Figure 1. **B.** Average gene expression of DNA-damage related genes on IMEC-WT and M2 cells. **C**. Cell cycle analysis of ER+ (MCF7), TNBC (BT549), TNBC $BRCA^{mut}$ (HCC1937) BC cell lines and BCSphCs (#1, #2, #3, #4, #5, #7, #8, #10, #11, #12, #13,

#21). Data are represented as mean ± SD of three independent experiments. **D**. Representative Comet assay images of ER+ (MCF7), TNBC (BT549) and TNBC $BRCA^{mut}$ (HCC1937) BC cell lines and BCSphCs (#21) treated with DOX (200nM) PTX (10nM) and CARB (100µM) for 24 hours (left panel) and relative tail length (right panel). A total of 100 nuclei were measured for each condition. Data are mean ± SD of three independent experiments. Scale bar represent 20 µm. **E.** Representative immunofluorescence analysis of γ-H2AX in ER+ (MCF7), TNBC (BT549) and TNBC $BRCA^{mut}$ (HCC1937) BC cell lines and BCSphCs (#21) treated with DOX (200nM) PTX (10nM) and CARB (100µM) for 4 hours (left panel) and relative quantization (right panel). Toto-3 counterstained nuclei. Data are mean ± SD of three independent experiments. Scale bar represent 30 µm. **F.** Clustergrams of up- and down-regulated genes involved in EMT and stemness in ER+ (MCF7) and TNBC (BT549) BC cell lines and BCSphCs (#4) treated with vehicle, doxorubicin (DOX), paclitaxel (PTX) and carboplatin (CARB) for 72 hours. **G.** Relative mRNA expression levels of *MYC* on ER+ (MCF7), TNBC (BT549) BC cell lines and BCSphCs (#4) treated as in D. Data are normalized to *GAPDH* expression levels and represented as fold mRNA level changes of treated over vehicle BC cells. **H.** Immunoblot analysis of Parp and Sam68 (input) and after immunoprecipitation (IP) with Sam68 antibody in TNBC (BT549) and TNBC $BRCA^{mut}$ (HCC1937). Lamin-B was used as loading control. **I.** Relative protein expression levels of Parp and Sam68 in the indicated BC cells. Data are mean ± SD (n=3). **J.** Relative protein expression levels of PAR, PARP and Sam68 in the indicated BC cells. Data are mean ± SD (n=3). **K.** Flow cytometry analysis of scramble (scr) and short hairpin Sam68 (shSam68) ER+ (MCF7), TNBC (BT549), TNBC $BRCA^{mut}$ (HCC1937) BC cells and BCSphCs (#3, #13, #21) transduced with the pDR-GFP reporter and SceI plasmids and treated with vehicle, DOX, PTX and CARB for 24 hours to monitor homologous recombination capacity. Data are represented as fold increase over vehicle. Data are mean ± SD (n=3).

Supplementary Figure 4. Sam68 and Rad51 inhibition hampered the growth of BCSphCs

A. Cell viability percentage of scramble (scr) and short hairpin Sam68 (shSam68) BCSphCs (#4, #13, #21) treated with vehicle and RI-1 (20µM) or BO2 (10µM), for 6 days. Data are represented as mean ± SD (n=3). **p-value≤0.01, ****p-value≤0.0001. **B.** Size of tumors generated by orthotopic injection of scramble (scr) and short hairpin Sam68 (shSam68) TNBC $BRCA^{mut}$ treated with vehicle (veh) and BO2. Arrows indicate the start and the end of treatment. Data are expressed as mean of tumors generated by the injection of TNBC $BRCA^{mut}$ ± SEM (n=5 mice per group). **C**. Cell viability percentage of BCSphCs (#4, #13, #21) treated with vehicle, olaparib (10µM), alone or in combination with RI-1 (20µM) or BO2 (10µM), for 6 days. Data are represented as mean ± SD

(n=3). **D.** Size of tumors generated by orthotopic injection of scramble (scr) and short hairpin Sam68 (shSam68) TNBC *BRCA*^{mut} treated with vehicle, olaparib, BO2, cisplatin and olaparib *plus* BO2 and olaparib *plus* cisplatin and BO2. Arrows indicate the start and the end of treatment. Data are expressed as mean of tumors generated by the injection of TNBC *BRCA*^{mut} ± SEM (n=5 mice per group). **E-H**. Kinetics of body weight oscillation in mice treated for three weeks as in Figure 4C,D and Supplementary Figure 4C,D. Time "0" indicate the start of treatment. Intermittent lines indicate the tolerable reduction in body weight, calculated as the reduction of 20% of mice weight at the beginning of treatment. **I**. Immunoblot analysis of Rad51 in TNBC *BRCA*^{mut} (HCC1937) BC cell lines treated with vehicle and dinaciclib for 24 hours at the indicated concentration. β-actin was used as loading control. **J.** Relative protein expression levels of Rad51 in BCSphCs (#4, #13, #15, #17 and #21) and TNBC *BRCA*^{mut} BC cells. Data are mean ± SD (n=3). **K**. Relative mRNA expression levels of *BRCA1* in TNBC and TNBC *BRCA*^{mut} BC cells and BCSphCs (#4, #13 and #21) treated for 24 hours with dinaciclib (10nM). GAPDH was used as housekeeping control gene. Data are represented as mean ± SD of three independent experiments. **L.** Cell viability percentage of scramble (scr) and short hairpin Sam68 (shSam68) TNBC *BRCA*^{mut} BC cell lines treated with vehicle and dinaciclib (10nM) for 6 days. Data are represented as mean of 2 independent experiments performed on MDA-MB-436 and HCC1937 cells ± SEM (n=2). *p-value≤0.05, ****p-value≤0.0001. **M**. Cell viability percentage of scramble (scr) and short hairpin Sam68 (shSam68) IMEC cell line treated with vehicle and dinaciclib (10nM) for 6 days. **N.** Size of tumors generated by orthotopic injection of scramble (scr) and short hairpin Sam68 (shSam68) TNBC *BRCA*^{mut} (MDA-MB-436 and HCC1937) BC cell lines treated with vehicle (veh) and dinaciclib (din). Arrows indicate the start and the end of treatment. Data are expressed as mean of tumors generated by MDA-MB-436 and HCC1937 ± SEM (n=5 mice per group). ****p-value≤0.0001. **O.** Representative immunohistochemical analysis of CD44, Sam68, γH2AX and Rad51 on paraffin-embedded sections of xenografts generated by HCC1937 cells as in (D) (*upper panels*). Representative RGB-3D color histograms representing the intensity and frequency of the colors showed in the upper panel (*lower panels*). Histograms represent the pixel intensity of the red color normalized to scramble vehicle (control). Data are represented as mean of 3 different fields (*lower right panels*). Scale bar represents 20 µm. **P.** Cell viability percentage of TNBC *BRCA*^{mut} (HCC1937) treated with vehicle, olaparib and dinaciclib, alone or in combination, at the indicated concentrations for 6 days. Data are represented as mean of 3 independent experiments ± SD. **Q.** Synergy plot representing the combination index (CI) for each olaparib and dinaciclib dose pair calculated from cell viability data of TNBC *BRCA*^{mut} (HCC1937) BC cell lines. **R.** Cell viability percentage of IMEC cells treated with vehicle and dinaciclib (10nM) for 6 days. Data are

represented as mean ± SD (n=3). **S.** Representative immunohistochemical analysis of Rad51 and γH2AX on paraffin-embedded sections of HCC1937 xenografts treated with vehicle, olaparib, dinaciclib and olaparib plus dinaciclib. Scale bar represents 20 µm. **T.** Size of tumors generated by orthotopic injection of TNBC *BRCA*^{mut} BC cell lines treated as in (P). Arrows indicate the start and the end of treatment. Data are expressed as mean of tumors generated with MDA-MB-436 and HCC1937 ± SEM (n=5 mice per group). ***p-value≤0.001. **U**. Representative H&E analysis on paraffin-embedded liver sections of mice engrafted with HCC1937 and treated with vehicle, olaparib, dinaciclib and olaparib plus dinaciclib. Lower panels magnification images. Scale bar represents 20 µm. **V**. Kinetics of body weight oscillation in mice treated for three weeks as in N. Time "0" indicate the start of treatment. Intermittent lines indicate the tolerable reduction in body weight, calculated as the reduction of 20% of mice weight at the beginning of treatment. **W**. Representative H&E analysis on paraffin-embedded liver sections of mice engrafted with HCC1937 and treated with vehicle, olaparib, dinaciclib and olaparib *plus* dinaciclib. Lower panels magnification images. Scale bar represents 20 µm. **X**. Kinetics of body weight oscillation in mice treated as in T. Intermittent lines indicate the tolerable reduction in body weight, calculated as the reduction of 20% of mice weight at the beginning of treatment.

Supplementary Figure 5. Sam68 and Rad51 inhibition affects proliferation of hormonal therapy-resistant breast cancer cells

A. Relative mRNA expression levels of *MYC* on ER+ and ER+^{R} (MCF7) BC cells. Data are represented as fold mRNA level changes of treated scr and shSam68 cells over vehicle. Data are represented as mean ± SD (n=3). **B**. Immunoblot analysis of Rad51 in ER+ and ER+^{R} (MCF7) BC cells treated with vehicle or dinaciclib for 24 hours at the indicated concentrations. β-actin was used as loading control (*left panel*). Relative band densities of immunoblots for Rad51 (*right panel*). **C**. Cell viability percentage of scramble (scr) and short hairpin Sam68 (shSam68) ER+ (MCF7) BC cell lines treated with vehicle or dinaciclib (10nM) for 6 days. Data are represented as mean ± SEM (n=4). ns, not significant, ***p-value≤0.001. **D.** Relative mRNA expression levels of *RAD51* and *MYC* on scramble (scr) and short hairpin Sam68 (shSam68) ER+ (MCF7) BC cells treated with vehicle or dinaciclib for 24 hours. Data are represented as fold mRNA level changes of treated scr and shSam68 over vehicle (n=3). **E.** Clustergrams of up- and down-regulated genes involved in stemness in scramble (scr) and short hairpin Sam68 (shSam68) ER+^{R} (MCF7) BC cell lines treated with vehicle and dinaciclib (10nM) for 6 days. **F.** Cell viability percentage in ER+ (MCF7) BC cells treated with vehicle, olaparib and dinaciclib, alone or in combination, at the indicated

concentrations for 6 days. Data are represented as mean ± SD (n=3). **G.** Synergy plot representing the combination index (CI) computed in CompuSyn by using Chou-Talalay method for each olaparib and dinaciclib dose pair calculated from cell viability data of ER+ and ER+^{R} (MCF7) BC cells. **H-I.** Kaplan-Meier plots of relapse-free survival (RFS) probability of BC patients of all molecular subtypes stratified by high or low *MYC* and (*BRCC5*) *RAD51* expression levels.

Supplementary Table 1. Description of breast cancer clinical features. ER =estrogen receptor; PR = progesterone receptor; HER2 = human epidermal growth factor receptor 2; TNBC = triple negative breast cancer; NA= not available.

Supplementary Table 2. *TP53* mutation status on BCSphCs. Intronic mutations and single-nucleotide polymorphism have been filtered out. Nucleotide variants were clinically validated in Clinvar or COSMIC database.

Supplementary Table 3. TP53 mutation status on breast cancer cells. Data retrieved from COSMIC Cell Lines Project v94 (released 28-MAY-21) compared with literature data (PMID:26067754; PMID:24700732)

Supplementary Table 4. Multivariate analysis of Sam68 expression in breast tumors.

Supplementary Table 5. Multivariate analysis of Sam68 expression in breast cancer subtypes.

Supplementary Table 6. Selected list of DNA repair genes retrieved from both public available databases and literature data.

Supplementary Table 7. TCGA BRCA and GTeX expression data (RNASeq2GeneNorm) of genes expressed in tumor *versus* normal breast tissues. Genes showing median expression levels ≥ 0.5 (log_2 fold-change) in breast cancer *versus* normal breast tissues were selected.

Supplementary Table 8. Gene set in the core enrichment of M2 cell of DNA damage repair gene enrichment analysis (GSEA).
