# Supplementary figures and images for: Effective targeting of breast cancer stem cells by combined inhibition of Sam68 and Rad51
Source: Oncogene. 2022 Feb 25;41(15):2196–209. doi: 10.1038/s41388-022-02239-4 (PMC8993694; doi:10.1038/s41388-022-02239-4)

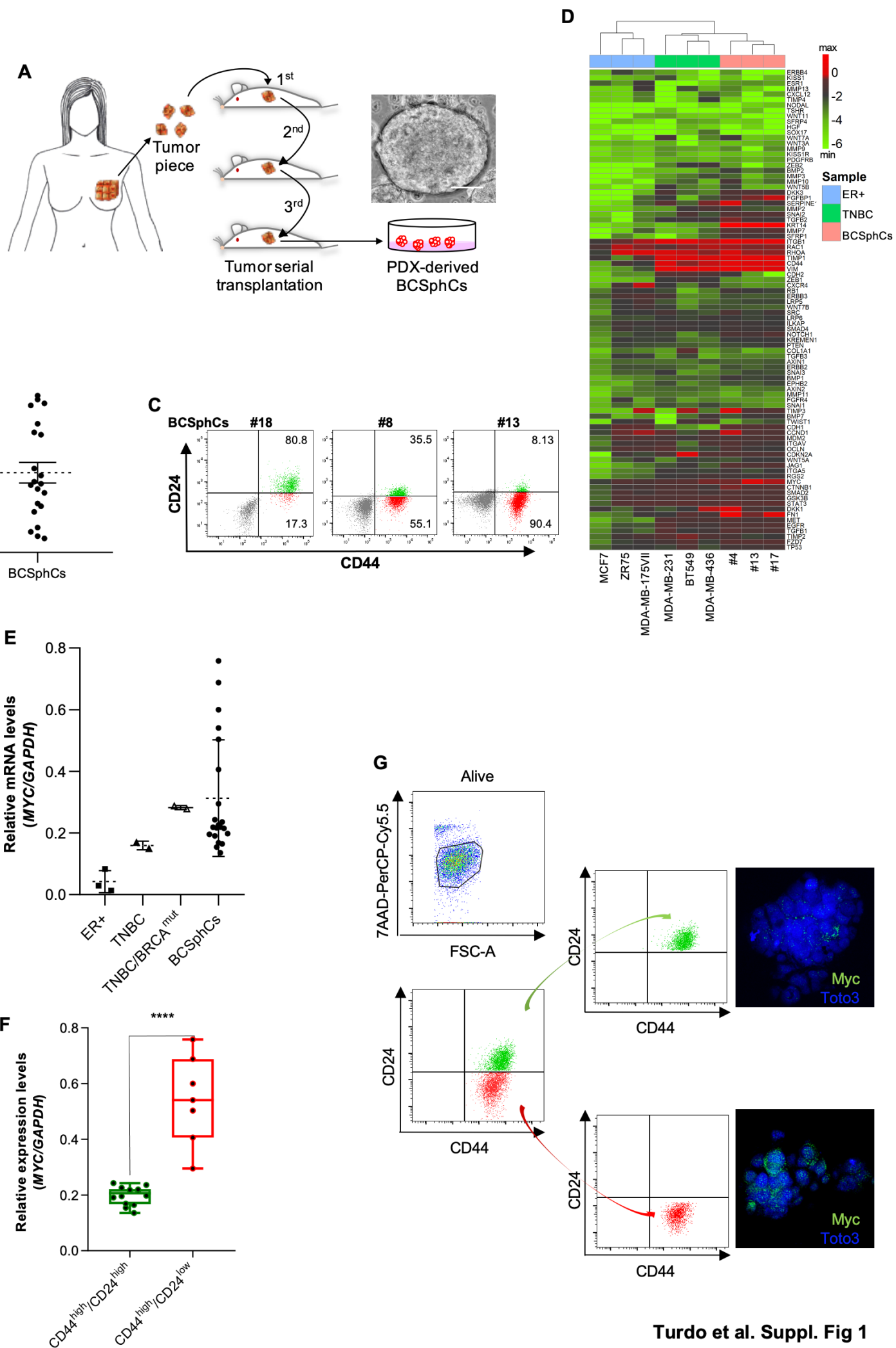

Supplement: Supplementary file 2 — Supplementary Figure 1 [file 41388_2022_2239_MOESM2_ESM.pdf]

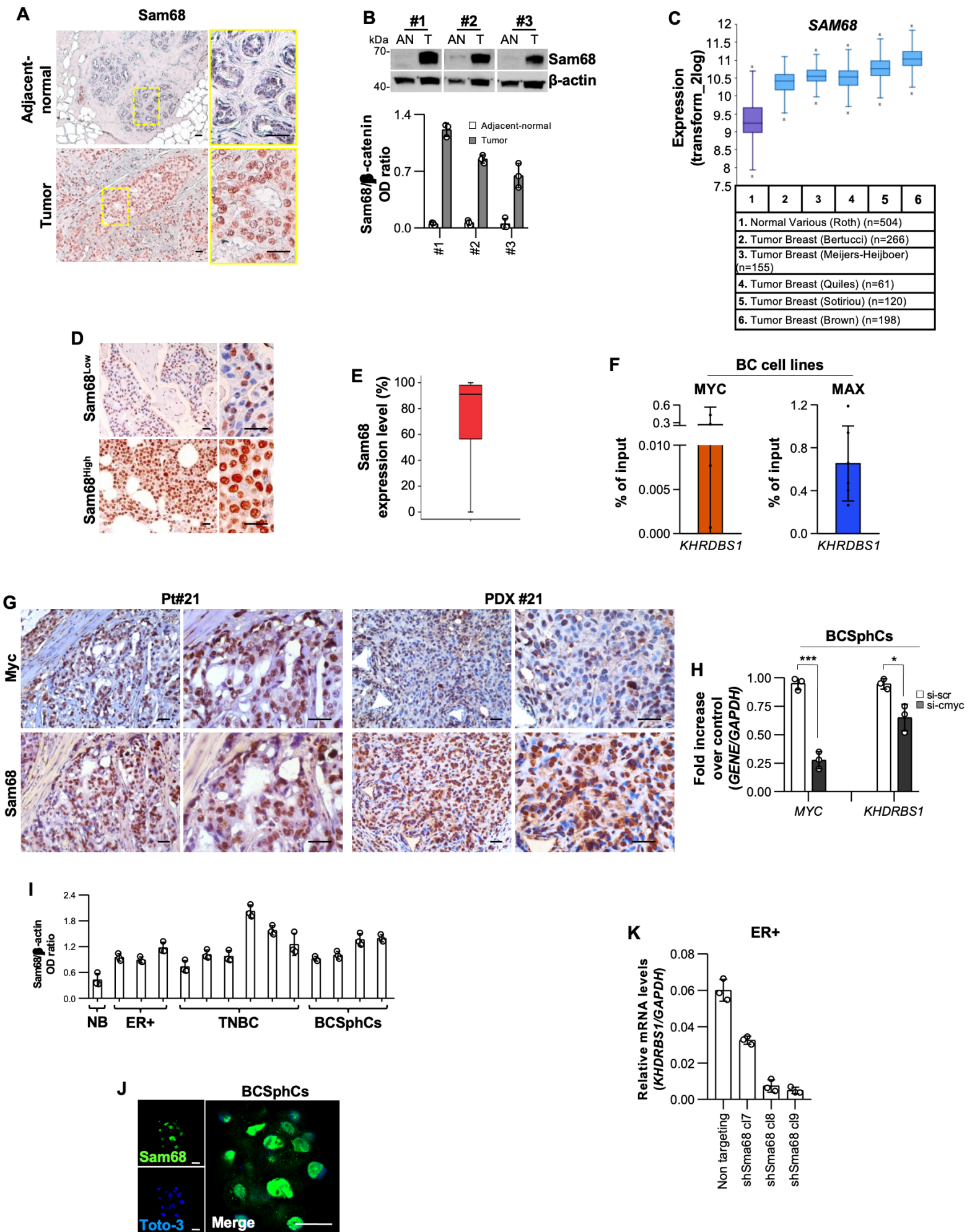

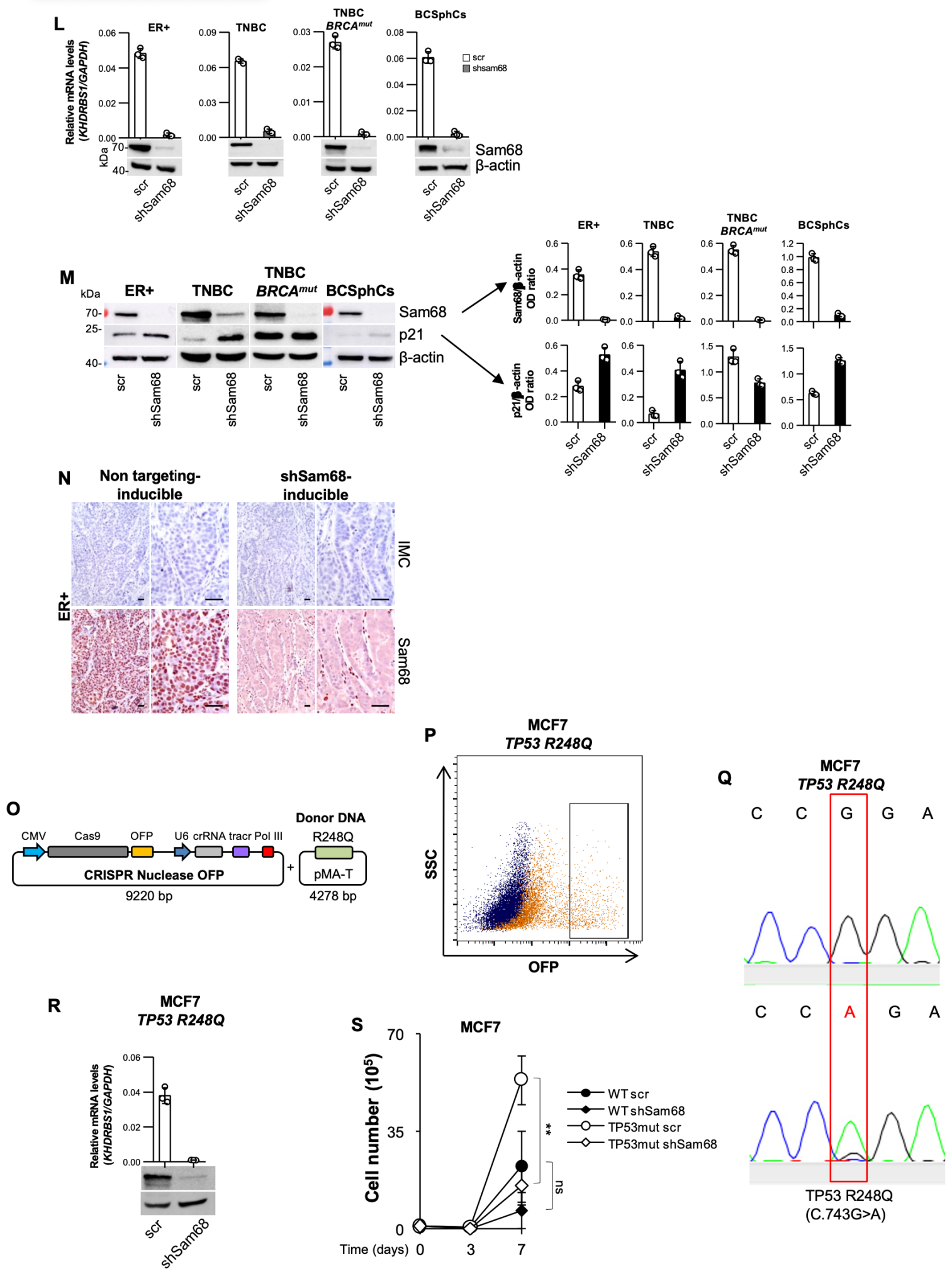

Supplement: Supplementary file 3 — Supplementary Figure 2 [file 41388_2022_2239_MOESM3_ESM.pdf]

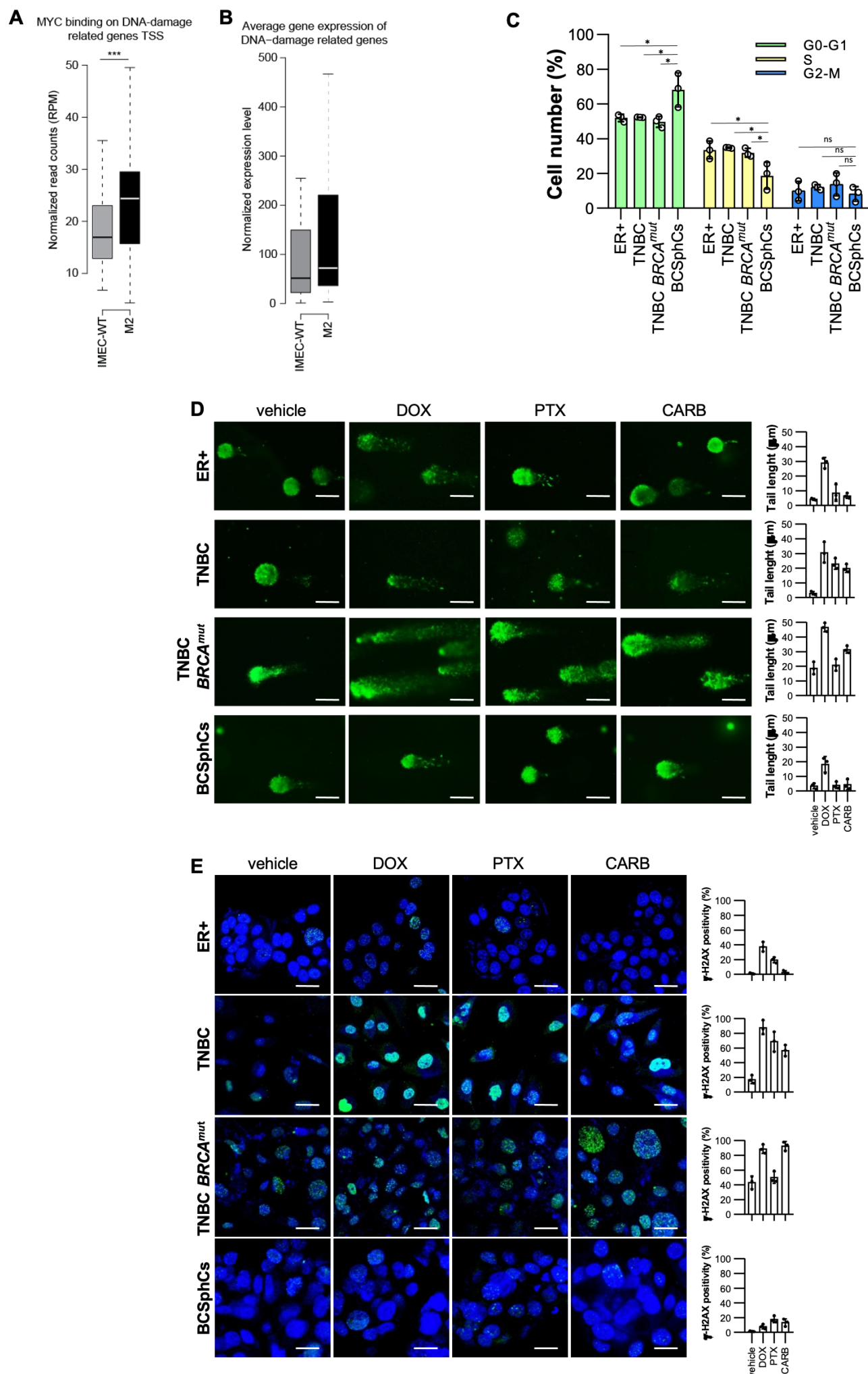

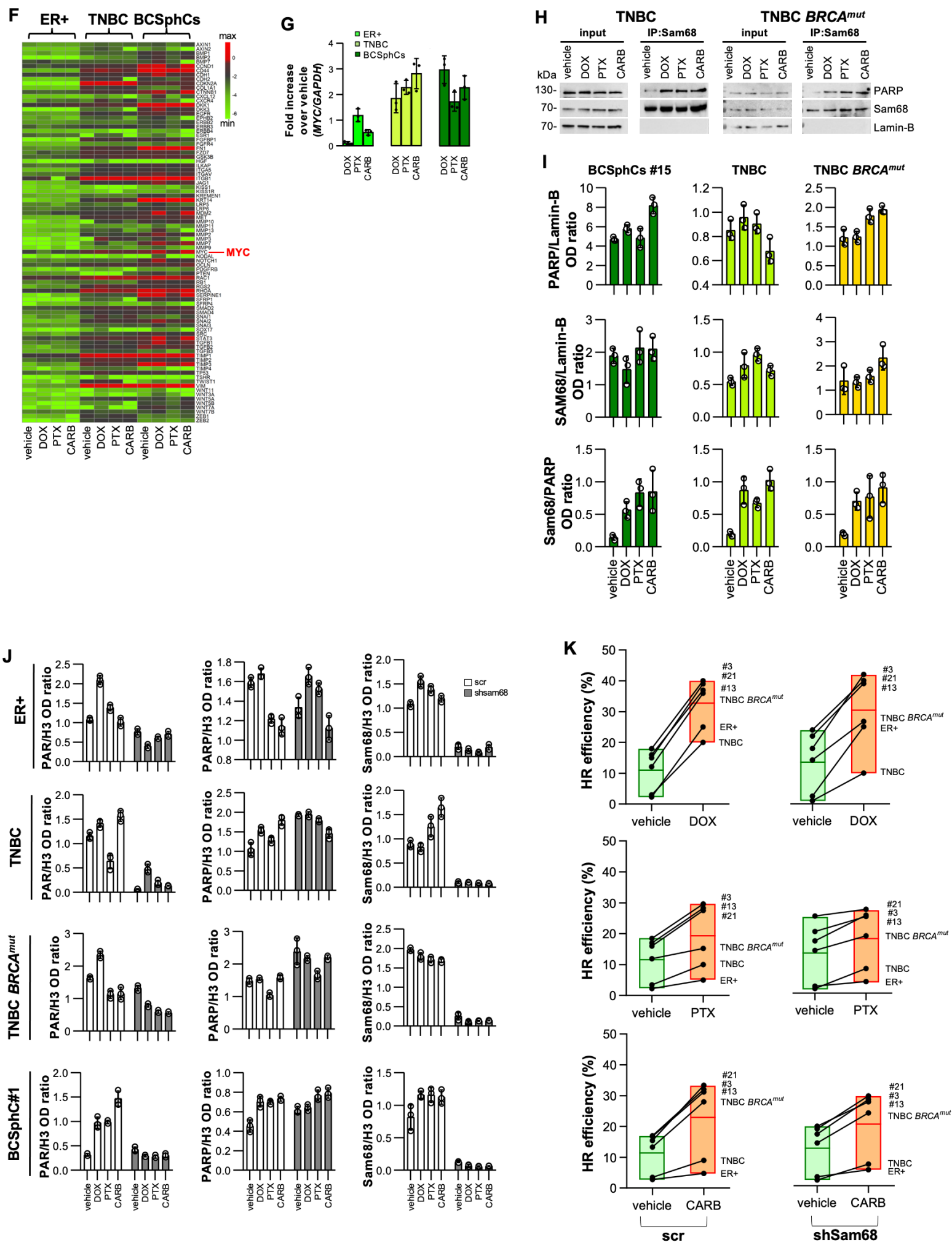

Supplement: Supplementary file 4 — Supplementary Figure 3 [file 41388_2022_2239_MOESM4_ESM.pdf]

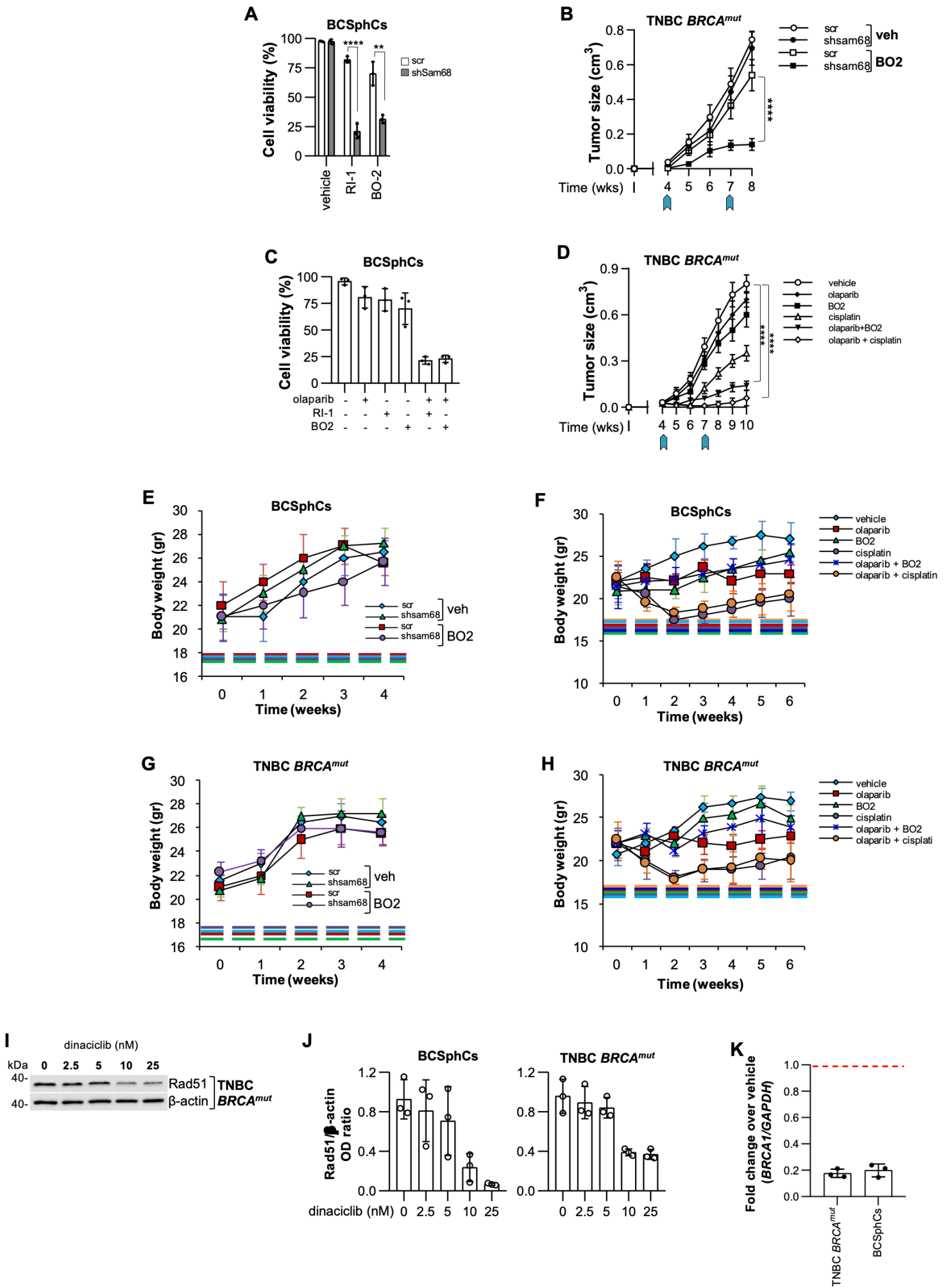



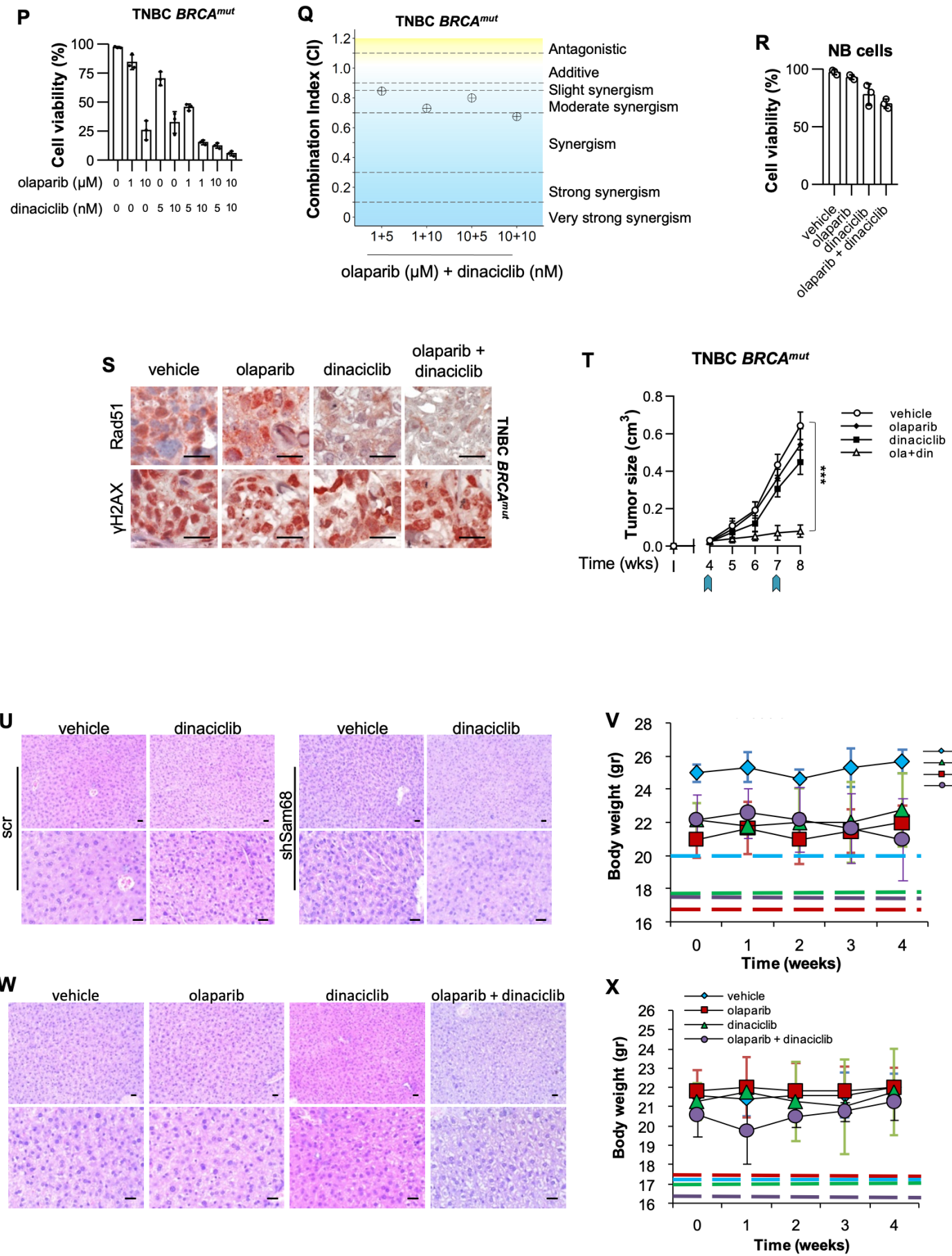

Supplement: Supplementary file 5 — Supplementary Figure 4 [file 41388_2022_2239_MOESM5_ESM.pdf]

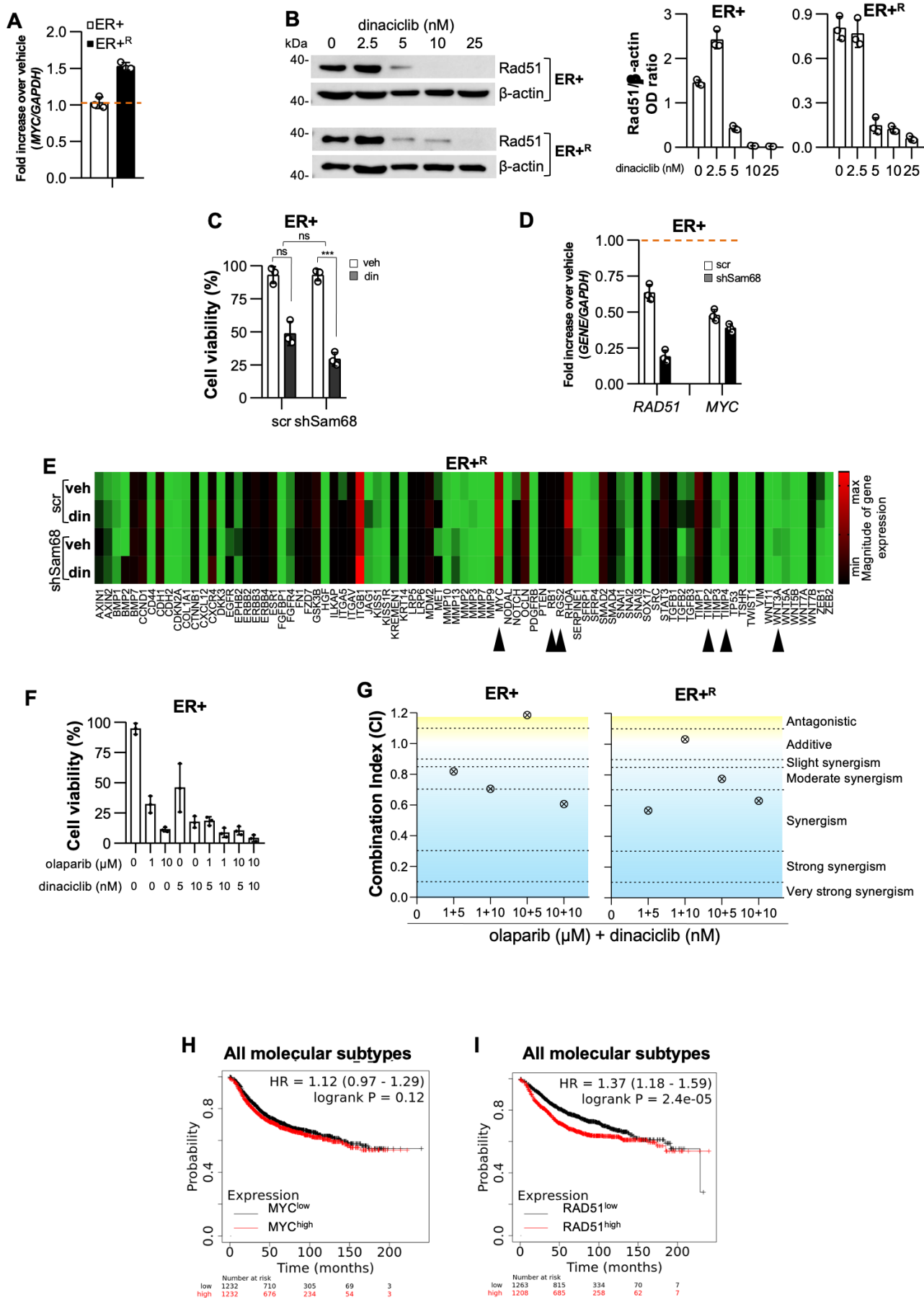

Supplement: Supplementary file 6 — Supplementary Figure 5 [file 41388_2022_2239_MOESM6_ESM.pdf]
